# Effects of a clinical medication review focused on personal goals, quality of life, and health problems in older persons with polypharmacy: A randomised controlled trial (DREAMeR-study)

**DOI:** 10.1371/journal.pmed.1002798

**Published:** 2019-05-08

**Authors:** Sanne Verdoorn, Henk-Frans Kwint, Jeanet W. Blom, Jacobijn Gussekloo, Marcel L. Bouvy

**Affiliations:** 1 Division of Pharmacoepidemiology & Clinical Pharmacology, Utrecht Institute for Pharmaceutical Sciences (UIPS), Utrecht University, Utrecht, The Netherlands; 2 SIR Institute for Pharmacy Practice and Policy, Leiden, The Netherlands; 3 Department of Public Health and Primary Care, Leiden University Medical Centre, Leiden, The Netherlands; 4 Department of Internal Medicine, Gerontology and Geriatrics Section, Leiden University Medical Centre, Leiden, The Netherlands; Harvard University, Brigham and Women's Hospital, UNITED STATES

## Abstract

**Background:**

Clinical medication reviews (CMRs) are increasingly performed in older persons with multimorbidity and polypharmacy to reduce drug-related problems (DRPs). However, there is limited evidence that a CMR can improve clinical outcomes. Little attention has been paid to patients’ preferences and needs. The aim of this study was to investigate the effect of a patient-centred CMR, focused on personal goals, on health-related quality of life (HR-QoL), and on number of health problems.

**Methods and findings:**

This study was a randomised controlled trial (RCT) performed in 35 community pharmacies and cooperating general practices in the Netherlands. Community-dwelling older persons (≥70 years) with polypharmacy (≥7 long-term medications) were randomly assigned to usual care or to receive a CMR. Randomisation was performed at the patient level per pharmacy using block randomisation. The primary outcomes were HR-QoL (assessed with EuroQol [EQ]-5D-5L and EQ-Visual Analogue Scale [VAS]) and number of health problems (such as pain or dizziness), after 3 and 6 months. Health problems were measured with a self-developed written questionnaire as the total number of health problems and number of health problems with a moderate to severe impact on daily life. Between April 2016 and February 2017, we recruited 629 participants (54% females, median age 79 years) and randomly assigned them to receive the intervention (*n* = 315) or usual care (*n* = 314). Over 6 months, in the intervention group, HR-QoL measured with EQ-VAS increased by 3.4 points (95% confidence interval [CI] 0.94 to 5.8; *p* = 0.006), and the number of health problems with impact on daily life decreased by 12% (difference at 6 months −0.34; 95% CI −0.62 to −0.044; *p* = 0.024) as compared with the control group. There was no significant difference between the intervention group and control group for HR-QoL measured with EQ-5D-5L (difference at 6 months = −0.0022; 95% CI −0.024 to 0.020; *p* = 0.85) or total number of health problems (difference at 6 months = −0.30; 95% CI −0.64 to 0.054; *p* = 0.099). The main study limitations include the risk of bias due to the lack of blinding and difficulties in demonstrating which part of this complex intervention (for example, goal setting, extra attention to patients, reducing health problems, drug changes) contributed to the effects that we observed.

**Conclusions:**

In this study, we observed that a CMR focused on personal goals improved older patients’ lives and wellbeing by increasing quality of life measured with EQ-VAS and decreasing the number of health problems with impact on daily life, although it did not significantly affect quality of life measured with the EQ-5D. Including the patient’s personal goals and preferences in a medication review may help to establish these effects on outcomes that are relevant to older patients’ lives.

**Trial registration:**

Netherlands Trial Register; NTR5713

## Abstract 2

In a randomised controlled trial, Sanne Verdoorn and co-workers assess a patient-centred clinical medication review and health-related quality of life in people aged ≥70 years living in the Netherlands

## Author summary

**Why was this study done?:**

- The number of older persons with long-term medication use will increase in the next decades.
- There is ample evidence that a medication review reduces drug-related problems but there is limited evidence whether this translates into an improvement of older persons’ wellbeing.
- We hypothesised that a medication review focused on patients’ health-related goals and preferences could improve their wellbeing.

**What did the researchers do and find?:**

- We conducted a pragmatic randomised controlled trial among 629 persons aged over 70 and using at least 7 long-term medications, comparing a clinical medication review in which participants established personal goals versus usual care.
- Participants were followed for 6 months, and we measured their quality of life and 12 different health problems, such as pain, dizziness, and dyspnoea, through written questionnaires at baseline and at 3 and 6 months.
- We found that a medication review in which the patient interview was focused on the patient’s preferences and goals improved older persons’ self-reported quality of life (EQ-VAS) and reduced health problems compared to usual care, although the quality of life measured with EQ-5D did not change.

**What do these findings mean?:**

- In our study, there was no effect on quality of life measured with EQ-5D, but other patient-reported outcomes, including quality of life measured with EQ-VAS, did improve, underlining the complexity of measuring improvement in the wellbeing of older persons.
- Focusing on the patient’s preferences during a medication review enables shared decision-making about pharmacotherapy in this older population.
- Future studies are needed to further assess the effects of including personal goals during medication review and to optimise and implement the use of such personal goals in care for older persons.

## Introduction

Medication reviews are increasingly performed and recommended by guidelines for older persons with multimorbidity and long-term medication use [1,2]. There are different types of medication reviews, ranging from a prescription review (which is basically an evaluation of the list of prescribed medicines) to a clinical medication review (CMR; with the availability of all clinical data and an extensive patient interview) [3]. It has been established that a CMR can identify and reduce drug-related problems (DRPs) and can have positive effects on other intermediate outcomes, such as low-density lipoprotein (LDL) cholesterol or HbA1c [4–11]. However, evidence for the effect of CMR on clinical outcomes, for example, hospital admissions and health-related quality of life (HR-QoL), is limited [6,7,12,13].

Several factors may contribute to the lack of clear evidence regarding the effect of CMR on clinical outcomes. First, the selection criteria for persons invited for medication review may have been too broad, for example, in participants aged ≥65 years and using ≥5 long-term medications [5,8,14,15]. A large proportion of these community-dwelling older persons might have had a relatively good quality of life and a low probability for clinical events such as hospital admissions. Preventing hospital (re)admissions is a very relevant goal of medication review, especially in high-risk patients after a recent hospital stay [16]. However, hospitalizations are rare in primary care, where a focus on the general health problems of older persons may be more important. Furthermore, different types of medication reviews (for example, treatment review or CMR) are performed in different settings (for example, primary care, nursing homes, or hospital wards), implying that interventions performed during medication review are often heterogeneous [5,12,16–20]. For example, these interventions can vary from adding prophylactic medication (such as statins) to starting symptomatic treatment (such as analgesics) and discontinuing medicines that cause side effects (for example, psychoactive drugs that cause dizziness or falling). This complicates measurement of any effect on a generic outcome, for example, quality of life. Finally, earlier studies may have involved healthcare providers without sufficient training or experience in a patient-centred CMR [21–23]. Previous studies have recommended that future studies investigating CMR should include high-risk patients and use relevant patient-related outcome measures [6,7,24,25].

In the DREAMeR (Drug use Reconsidered in the Elderly using goal Attainment scales during Medication Review) study, we developed a patient-centred approach to CMR in which the health problems, preferences, and personal goals of the older person receive specific attention. The aim of the present randomised controlled trial (RCT) was to determine the impact of such a patient-centred approach in CMR on patients' lives, including HR-QoL and health problems.

## Methods

### Study design and setting

The DREAMeR study was a pragmatic RCT performed in 35 community pharmacies in the Netherlands, comparing a CMR focused on personal goals with usual care. The design, conduct, and reporting of DREAMeR adhere to the Consolidation Standards of Reporting Trials (CONSORT) guidelines (S1 CONSORT Checklist). Between April 1st, 2016, and February 28th, 2017, patients were invited by their pharmacists to participate in this study, and participants were followed for 6 months. Randomisation of participants to the intervention or control group was carried out at patient level and performed after recruitment of the participants. Block randomisation per pharmacy using a computer-generated list of random numbers was applied by the researcher (SV) to obtain equal numbers of persons per pharmacy per group. A block consisted of the number of patients who agreed to participate at each pharmacy. Participation was voluntary, and all participants provided signed informed consent. The nature of the intervention made blinding impossible. The full study protocol has been published elsewhere (S1 Study Protocol) [26].

### Study participants

Persons were eligible for inclusion in the study if they were aged ≥70 years and using ≥7 long-term medications. These criteria were stricter than the criteria used in most studies (≥65 years and using ≥5 long-term medications) because we expected that increasing age and number of medications would identify more frail patients with more complex diseases. Exclusion criteria were i) an expected life expectancy of ≤6 months, ii) hospital admission within one month before the inclusion date, iii) having received a CMR in the past 12 months, and iv) receiving repeat prescriptions solely from a hospital specialist. In addition to these criteria, general practitioners (GPs) made an estimation of the patient’s ability to participate in this study, which may have led to exclusion of patients with cognitive impairment. Participants were recruited by their community pharmacists. First, the pharmacists screened all their patients by the inclusion criteria, then sent the list of the selected patients to the patients’ GPs. The GPs judged the patients according to the exclusion criteria. An anonymised list was then sent to the researcher (SV) to randomly assign 50 patients, who were invited first. These patients were subsequently invited to participate by letter and/or telephone consultation by their pharmacist. If the first random sample did not include enough patients, another group of 50 patients was invited.

### Community pharmacists and GPs

The study was conducted at Dutch community pharmacy franchises of ‘Service Apotheek’. They were recruited within this population of pharmacists based on the following selection criteria: participating community pharmacists had to be accredited to perform CMR and should have performed at least 25 CMRs annually over the past 3 years. Finally, they should have an agreement with at least one GP to join CMR. The participating pharmacists received 1-day training about all aspects of the study, for example, registration, data collection, communication skills, and goal setting in older persons during CMR. Each pharmacist collaborated in the CMRs in this study with at least one GP; all GPs were informed by the pharmacists about the study. During the study, monthly web conferences were organised and chaired by a member of the research team, in which study progress and CMR cases were discussed. Every participating pharmacist in this study attended the web conferences and presented one case of a CMR.

### Intervention

Patients in the intervention group received a CMR review focused on personal goals. The multidisciplinary guideline ‘Polypharmacy in the Elderly’ in the Netherlands was followed to perform these CMRs [2]. Full drug-dispensing records from the pharmacy and clinical records from the general practice (including disease history and laboratory values) were available at the start of the CMR. The process of CMR consisted of 5 different steps: 1) A patient interview performed by the community pharmacist, consisting of an extensive discussion of the patient’s health problems, patient’s preferences, and all medications currently used, including over-the-counter (OTC) medication (addressing the effectiveness, usage, adherence, side effects, and practical problems associated with all the medications). At the end of the interview, all the problems were summarised, and one or more health-related goals were proposed. These goals could be related to the patient’s health problems or other wishes related to medication and disease and were (as far as possible) SMART (specific, measurable, acceptable, realistic, and time-bound) formulated. 2) After the patient interview, all potential DRPs were summarised by the pharmacist, and recommendations were proposed to attain goals and to solve DRPs. 3) The pharmacist had a face-to-face meeting with the patient’s GP to discuss all health-related goals and other identified DRPs. They then proposed a pharmaceutical care plan, including which actions should be carried out, as well as when and by whom. 4) The pharmaceutical care plan was then discussed with the patient to reach agreement about implementation. 5) Two follow-up appointments were scheduled (within approximately 3 months), in which the pharmacist evaluated the agreed actions and the attainment of goals with the patient and, if necessary, adjusted the treatment plan after discussion with the GP. The CMR was expected to be completed within approximately 3 months, depending on the type of interventions.

The contribution of the patient was ensured by using the questionnaires on health problems at the start of the CMR as input for the pharmacist and by having the patient propose their personal health-related goals during the patient interview. A novel, to our knowledge, aspect of the present study was that goals were proposed during the patient interview and evaluated using goal attainment scaling (GAS). GAS is an individualised goal-setting and measurement approach that is useful for patients with multiple, individualised health problems [27,28]. The scores on GAS at 3 and 6 months were independently collected by research assistants during telephonic interviews with the participants in the intervention group.

### Usual care

The patients in the control group received usual care and were placed on a waiting list; all of them were offered a CMR after the end of the study (postponed intervention). In the Netherlands, community pharmacists register all dispensed prescription medicines in their pharmacy information system, and patients generally receive their prescribed medication from one pharmacy and visit one GP. Pharmacy and GP information systems frequently exchange information. The electronic patient record in these systems includes data on dispensed medications, coded chronic diseases, and laboratory values. Prescriptions can be sent electronically from physician to pharmacy (for most GPs). OTC medication can also be obtained from so-called druggists. Information on OTC medication is frequently missing in both GP and pharmacy systems. Clinical decision support with drug therapy alerts, including drug–drug interactions and drug–disease interactions, is an integral part of the pharmacy information system [29]. Because of their legal and professional shared responsibility for the safe and effective use of medication, pharmacists regularly contact prescribers regarding prescribing issues. The majority of GPs and community pharmacists regularly discuss prescribing guidelines in pharmacotherapy audit meetings. Since the publication of the multidisciplinary guideline ‘Polypharmacy in the Elderly’, more and more pharmacists and GPs work together in the field of CMRs. A CMR is not offered to all patients, but the majority of pharmacies offer medication review yearly to a proportion of their older patients [2].

### Outcome measures

The primary outcome measures were i) HR-QoL measured with the EuroQol (EQ)-5D-5L and the EQ-Visual Analogue Scale (VAS) (range 0 to 100) and ii) the number of health problems. These outcome measures were collected through written questionnaires at baseline, 3 months, and 6 months. Questionnaires were sent to patients by the pharmacists but completed independently by the patients. If in need of assistance, patients could obtain help from an independent research assistant. All questionnaires were captured in duplicate by 2 independent research assistants who were blinded to the allocation of the patient. Health utility values (range −0.329 to 1) were calculated for EQ-5D-5L, indicating (less than) 0 as death and 1 as best possible health status [30]. An additional cognition-question (EQ-6D) was included in the questionnaire but was not used to calculate health utility values because, currently, no tariff exists for the EQ-6D [31]. Health problems were divided into total problems per patient (irrespective of the severity) and number of health problems per patient with a moderate to severe impact on daily life. A list of 12 health problems was measured based on the most commonly reported health problems in older people and the most common side effects of drugs (S1 Table and S1 Fig) [32]. We assessed each of the health problem’s severity and impact on the patient’s daily life. We defined a health problem with impact on daily life as one with a severity score of ≥5 on a VAS (range 0–10) [33] and moderate, severe, or extreme influence on daily life (i.e., ≥3 points on a 5-point Likert scale).

Secondary outcome measures were i) number of long-term medications, ii) number of prescribed drugs added and ceased, iii) severity of complaints measured with VAS scores (range 0–10), and iv) healthcare consumption. Medication was considered as long-term medication use if it was dispensed more than three times in the 12 months prior to the index date or for a consecutive period of at least 90 days prior to index date. Healthcare consumption was measured with the Dutch Medical Consumption (iMTA) Questionnaire, including an extra question about informal care through patient interviews by telephone performed by independent study assistants [34]. In the intervention group, process outcomes measured during the CMRs were number of health-related goals, attainment of goals measured with GAS, and number of identified and solved DRPs per patient. Drug-dispensing records were used to determine the number and type of long-term medications in use during each month. Finally, demographic information was collected, including the Integrated Systematic Care for Older People (ISCOPE) questionnaire, to determine complex health problems in the participants [35].

### Sample size

Calculation of a sample size was performed based on a change in health utility values of the EQ-5D from 0.05 with a standard deviation (SD) of 0.20 over 6 months; this difference was considered to be clinically relevant and feasible, based on previous studies [10,36]. This difference corresponded with a 5-point change in EQ-VAS. To achieve a statistically significant difference in the utility on the EQ-5D with alpha = 0.05 and beta = 0.20, a group size of 252 patients was sufficient. When correcting for a potential drop-out of 25%, a total of 630 patients was needed (i.e., 315 per group).

### Statistical analyses

Descriptive statistics were used to describe patient characteristics. Differences in drop-out between both groups were tested with an independent sample *t* test. The analysis was based on intention to treat, and effects of the intervention on outcome measures over 6 months were estimated using linear mixed model analyses (S1 Statistical Analysis Plan). Intervention, time (baseline and at 3 and 6 months), and the interaction between intervention and time were entered as fixed factors in the model. For effects on the number of long-term medications, the intervention, time (per month), and interaction between intervention and time were entered as fixed factors in the model. Participant identification number was included as a random effect to account for the dependence of repeated observations. Adjustment for sex, age, and pharmacy was made in the final models. Finally, a per protocol analysis was performed. For the per protocol analysis, the patients in the control group who accidentally received a CMR during the study period were excluded. Data were analysed using IBM SPSS Statistics 24.0 (IBM Corporation, Armonk, NY, USA). A *p*-value < 0.05 was considered statistically significant.

### Ethical statement

This study was approved by the Medical Ethics Committee of the University Medical Centre of Utrecht (protocol number METC 15–737). Written informed consent from all participants was obtained prior to the start of the study. The trial was registered in the Netherlands Trial Register, number NTR5713.

## Results

**Fig 1:**
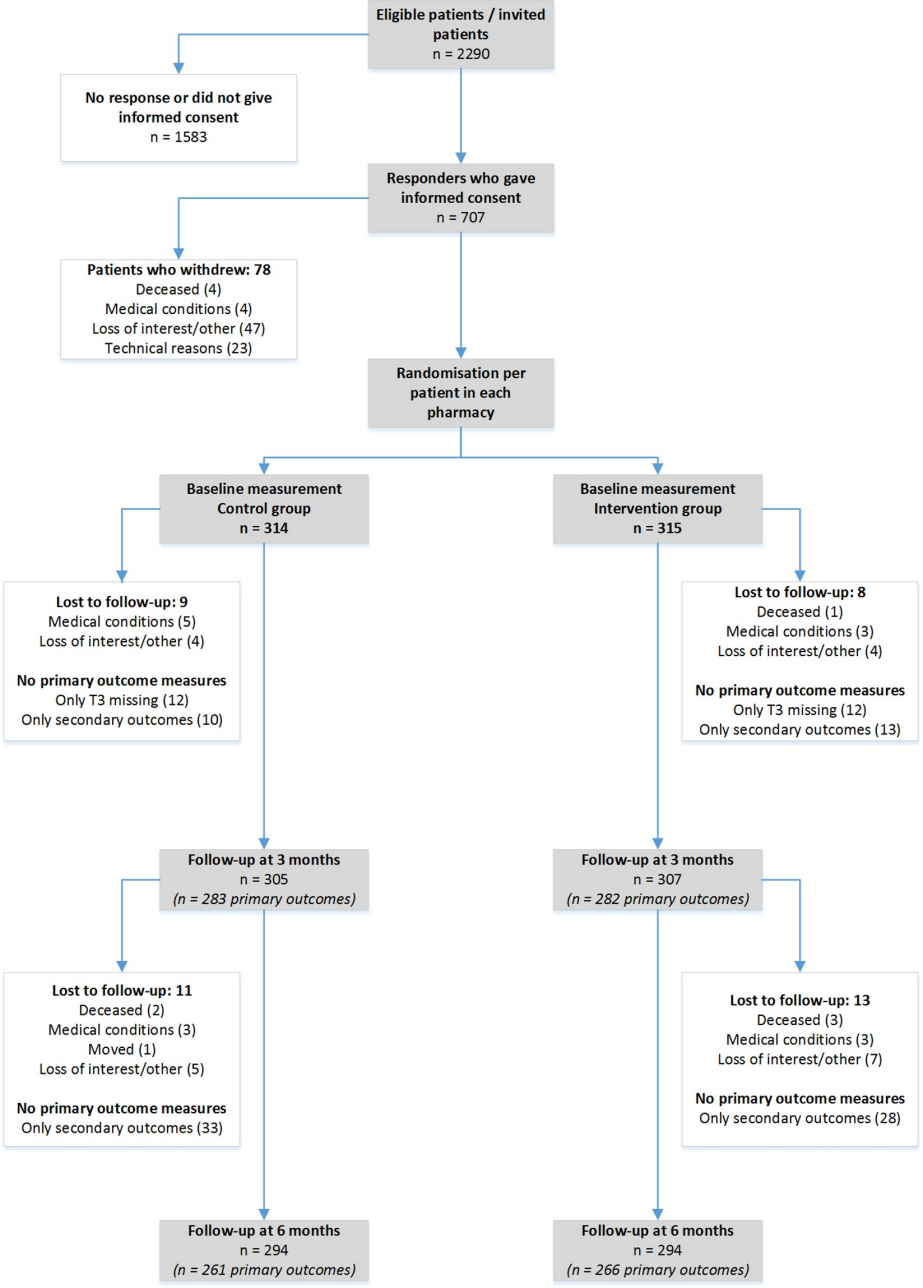
Flowchart of the study population.

Of the 2,290 invited patients, 707 (31%) consented to participate (Fig 1). Participants were recruited between April 2016 and February 2017; follow-up was performed up to August 2017.

A total of 629 patients were randomised: 314 into the control and 315 to the intervention group. Over 6 months, the total drop-out rates were similar in both groups (6.7% in the intervention group and 6.4% in the control group). In the control group, 2 patients died before the end of the study, compared to 4 patients in the intervention group. A total of 588 patients completed the study, and complete data for the primary outcomes were available from 503 patients. All patients in the intervention group received the intended treatment. Since 7 patients in the control group received a CMR before the end of the study, these patients were excluded from the per protocol analysis. The patient characteristics for the intervention and control group are shown in Table 1. The number of participants per pharmacy ranged from 2–30 (per pharmacy: mean 18, SD = 8). A total of 43 community pharmacists (working in 35 community pharmacies) and 113 GPs participated in this study. The average time spent by the community pharmacists to completely perform a CMR was 107 ± 40 minutes.

**Table 1:** Characteristics of participants in the control and intervention group.

Characteristic		Control group (*n* = 314)	Intervention group (*n* = 315)
**Sociodemographic**			
Age, median (IQR), years		78 (74–82)	80 (76–83)
Sex, female, ***n***/***N*** (%)		163/314 (52%)	176/315 (56%)
Ethnicity, European, ***n***/***N*** (%)		308/314 (98%)	306/315 (97%)
Living situation, alone, ***n***/***N*** (%)		116/314 (37%)	139/315 (44%)
Complex health problems†, ***n***/***N*** (%)		75/314 (24%)	79/315 (25%)
**Drug-related**			
Number of long-term medications, median (IQR)		9.0 (7.5–10.5)	9.0 (7.5–10.5)
Multidose Drug Dispensing system use, ***n***/***N*** (%)		69/314 (22%)	85/315 (27%)
**Top 10 Drug classes (ATC4 level), *n***/***N (%)***			
A02B	Drugs for peptic ulcer and GORD	254/314 (81%)	261/315 (83%)
B01A	Antithrombotic agents	245/314 (78%)	249/315 (79%)
C10A	Lipid-modifying agents	232/314 (74%)	224/315 (71%)
C07A	Beta-blocking agents	220/314 (70%)	189/315 (60%)
C08C	Selective calcium channel blockers	119/314 (38%)	101/315 (32%)
A10B	Oral blood-glucose–lowering drugs	110/314 (35%)	98/315 (31%)
C09A	Ace inhibitors	119/314 (38%)	95/315 (30%)
C09C	Angiotensin II antagonists	94/314 (30%)	91/315 (29%)
A11C	Vitamin D	88/314 (28%)	79/315 (25%)
C03C	High-ceiling diuretics	72/314 (23%)	72/315 (23%)

†Complex health problems measured with ISCOPE score.

**Abbreviations**: ATC, Anatomical Therapeutic Chemical classification; GORD, gastro-oesophageal reflux disease; IQR, interquartile range; ISCOPE, Integrated Systematic Care for Older People.

### Primary outcomes

Table 2 shows the unadjusted scores for primary outcomes at baseline and at 3 and 6 months. HR-QoL measured with the EQ-5D-5L showed no significant difference over time between the intervention and control group. The effect at 3 months was −0.0011 (95% confidence interval [CI] −0.012 to 0.010; *p* = 0.85; Table 3), and the effect at 6 months was −0.0022 (95% CI −0.024 to 0.020; *p* = 0.85) for the intervention group compared to the control group. HR-QoL measured with the EQ-VAS improved over time in the intervention group compared to the control group: the effect at 3 months was an increase of 1.7 points (95% CI 0.47 to 2.9; *p* = 0.006; Table 3), and the effect at 6 months was an increase of 3.4 points in quality of life (95% CI 0.94 to 5.8; *p* = 0.006) for the intervention group compared to the control group. Unadjusted regression scores showed similar results (S2 Table). The scores on the six domains of the EQ-6D showed no differences between the two groups (S3 Table).

**Table 2:** Unadjusted scores for HR-QoL and health problems over time in the control and intervention group.

	Control group			Intervention group		
Outcome (mean, SD)	Baseline (*n* = 314)	T1 = 3 months (*n* = 283)	T2 = 6 months (*n* = 261)	Baseline (*n* = 315)	T1 = 3 months (*n* = 282)	T2 = 6 months (*n* = 266)
**HR-QoL**						
EQ-5D-5L, utility values	0.74 (0.18)	0.74 (0.17)	0.74 (0.18)	0.73 (0.18)	0.74 (0.18)	0.73 (0.20)
EQ-VAS	70 (16)	69 (16)	69 (15)	68 (16)	69 (17)	70 (16)
**Health problems**						
Total problems	5.5 (2.9)	5.3 (2.7)	5.3 (2.9)	5.9 (3.0)	5.6 (3.0)	5.5 (3.0)
Problems with impact	2.6 (2.4)	2.5 (2.3)	2.5 (2.4)	2.8 (2.4)	2.5 (2.4)	2.4 (2.4)

Definition of problem with impact is a severity VAS score ≥5 and influence on daily life: moderate, severe, extreme.

**Abbreviations**: EQ, EuroQol; HR-QOL, health-related quality of life; SD, standard deviation; VAS, Visual Analogue Scale.

**Table 3:** Main outcomes of the linear mixed model analysis for intervention group compared to control group for HR-QoL and health problems.

Outcome	Group		Time		Group * Time	
	β	95% CI	β	95% CI	β	95% CI
**HR-QoL**						
EQ-5D-5L, utility values	+0.0073	−0.025 to 0.040	−0.0024	−0.010 to 0.0054	−0.0011	−0.012 to 0.010
EQ-VAS	−3.2*	−6.3 to −0.010	−1.0*	−1.9 to −0.17	+1.7**	0.47 to 2.9
**Health problems**						
Total problems	+0.30	−0.14 to 0.74	−0.041	−0.16 to 0.081	−0.15	−0.32 to 0.027
Problems with impact	+0.11	−0.24 to 0.46	−0.012	−0.12 to 0.091	−0.17*	−0.31 to −0.022

β coefficient and 95% CI for group (control versus intervention group), time (per 3 months for HR-QoL and health problems), and group by time interaction (adjusted for age, sex, pharmacy). Definition of health problem with impact is a severity VAS score ≥5 and influence on daily life: moderate, severe, extreme. The estimators in the column ‘group * time’ show the main difference in effects between the intervention group versus control group per 3 months for HR-QoL and health problems.

*p < 0.05

**p < 0.01

**Abbreviations**: CI, confidence interval; EQ, EuroQol; HR-QoL, health-related quality of life; VAS, Visual Analogue Scale.

No significant differences between groups were found in the total number of health problems, irrespective of severity. The effect at 3 months was −0.15 per 3 months (95% CI −0.32 to 0.027; *p* = 0.099; Table 3) and the effect at 6 months was −0.30 (95% CI −0.64 to 0.027, *p* = 0.099) for the intervention group compared to the control group. Compared to the control group, in the intervention group, the number of health problems with a moderate to severe impact on daily life decreased over time. The effect at 3 months was a reduction of −0.17 health problems (95% CI −0.31 to −0.022; *p* = 0.024; Table 3), and the effect at 6 months was a reduction of −0.34 health problems (95% CI −0.62 to −0.044, *p* = 0.024) for the intervention group compared to the control group, which is a reduction of 12% compared to baseline. Per protocol analyses did not show different results compared to the intention-to-treat analyses (S4 Table).

### Secondary outcomes

Compared to the control group, in the intervention group, the total number of long-term medications decreased with −0.32 medications after 6 months (β = −0.054 per month; 95% CI −0.094 to −0.014; *p* = 0.008). A mean number of 1.7 (SD 1.7) drugs per patient was added in the intervention group compared to 1.4 (SD 1.7) drugs in the control group (*p* = 0.011); a mean number of 1.5 (SD 1.5) drugs was stopped in the intervention group compared to 1.0 (SD 1.3) in the control group (*p* < 0.001). The most frequently added and ceased drugs in both groups are shown in S5 Table. Changes in long-term medication use over time are shown in S2 Fig.

There was large variability in the prevalence and severity of the 12 health problems, with pain, mobility, and fatigue being the most prevalent in both groups (Table 4). No effects were found in frequencies and severities between the groups over time (Table 5).

**Table 4:** Baseline prevalence and severity measured with VAS score of the 12 different health problems, comparison between control and intervention group.

Type of health problem (*n*/*N*, %)	Control group (*n* = 314)			Intervention group (*n* = 315)		
	Total problems	Problems with impact	VAS score, mean (SD)	Total problems	Problems with impact	VAS score, mean (SD)
Pain	232/314 (74%)	135/314 (43%)	5.1 (2.1)	227/315 (72%)	129/315 (41%)	5.1 (2.2)
Itching	107/314 (34%)	44/314 (14%)	4.2 (2.2)	110/315 (35%)	35/315 (11%)	4.0 (2.3)
Dyspnoea	251/314 (48%)	79/314 (25%)	4.8 (2.2)	161/315 (51%)	72/315 (23%)	4.7 (2.1)
Mobility	242/314 (77%)	166/314 (53%)	5.7 (2.2)	246/315 (78%)	173/315 (55%)	5.9 (2.3)
Dizziness*	110/314 (35%)	35/314 (11%)	3.6 (2.0)	135/315 (43%)	50/315 (16%)	4.1 (2.2)
Sedation*	60/314 (19%)	20/314 (6.4%)	3.8 (2.0)	82/315 (26%)	25/315 (7.9%)	3.7 (2.1)
Intestinal problems	116/314 (37%)	53/314 (17%)	4.7 (2.2)	126/315 (40%)	63/315 (20%)	4.9 (2.2)
Stomach problems	72/314 (23%)	15/314 (4.8%)	3.5 (2.1)	72/315 (23%)	23/315 (7.3%)	3.9 (2.3)
Cognition	157/314 (50%)	27/314 (8.6%)	3.2 (1.8)	158/315 (57%)	35/315 (11%)	3.4 (2.1)
Fatigue	214/314 (68%)	126/314 (40%)	5.1 (2.2)	227/315 (72%)	139/315 (44%)	5.2 (2.1)
Dry mouth	135/314 (43%)	60/314 (19%)	4.6 (2.2)	145/315 (46%)	57/315 (18%)	4.6 (2.3)
Incontinence	119/314 (38%)	53/314 (17%)	4.5 (2.5)	135/315 (43%)	57/315 (18%)	4.6 (2.5)

Data on <5% were missing.

*Prevalence at baseline showed a significant difference between the groups for this health problem. Definition of health problem with impact is a severity VAS score ≥5 and influence on daily life: moderate, severe, extreme.

**Abbreviations**: SD, standard deviation; VAS, Visual Analogue Score.

**Table 5:** Effects in severity measured with VAS scores over time: comparison between intervention and control group for the 12 different health problems.

Type of health problem	Effects severity		
	β	95% CI	*p*-value
Pain	−0.075	−0.26 to 0.11	0.43
Itching	−0.10	−0.40 to 0.20	0.49
Dyspnoea	−0.21	−0.45 to 0.033	0.091
Mobility	−0.086	−0.26 to 0.089	0.34
Dizziness	−0.27	−0.57 to 0.029	0.077
Sedation	−0.20	−0.61 to 0.20	0.32
Intestinal problems	−0.18	−0.47 to 0.12	0.24
Stomach problems	+0.022	−0.34 to 0.39	0.90
Cognition	−0.021	−0.23 to 0.19	0.84
Fatigue	−0.17	−0.34 to 0.0024	0.053
Dry mouth	−0.17	−0.44 to 0.11	0.23
Incontinence	+0.067	−0.19 to 0.33	0.61

β coefficient and 95% CI group by time interaction (adjusted for age, sex, pharmacy). The estimators in the column ‘group * time’ show the main difference in effects between the intervention group versus control group per 3 months for the severity measured with VAS scores per health problem.

**Abbreviations**: CI, confidence interval; VAS, Visual Analogue Scale.

The healthcare consumption over the 6-month study period in both groups is shown in S6 Table.

### Process outcomes

At least one health-related goal was proposed in 283 patients (90%) in the intervention group, with a mean of 1.4 per patient (SD = 0.52). The goals ‘reduce pain’ (*n* = 66, 16%), ‘reduce number of pills’ (*n* = 57, 14%), and ‘improve mobility’ (*n* = 37, 9.1%) were most prevalent [37]. Of the total number of 406 proposed goals, 350 were evaluated with GAS in 256 patients at 3 months (86%) and 347 goals in 247 patients at 6 months (86%). At 3 and 6 months, 37% and 43% of the goals were achieved, respectively. The mean number of DRPs per patient was 5.8 (SD 2.1); of these DRPs, 67% were solved and led to an intervention. The different types of identified and solved DRPs are shown in S7 Table. Of all DRPs, 28% were related to a health-related goal.

## Discussion

In this study, we found that a CMR focused on personal goals improved quality of life measured by the EQ-VAS in older persons with polypharmacy and reduced the number of health problems with a moderate to severe impact on daily life. Concurrently, CMR decreased the number of long-term medications. However, CMR had no effect on HR-QoL measured by the EQ-5D-5L or total number of health problems.

To our knowledge, this is one of the first studies to demonstrate a beneficial effect of CMR on HR-QoL measured with the EQ-VAS and to show an effect on the number of health problems. Only one previous study has shown CMR to affect HR-QoL according to both utility values and VAS scores [36]; in that study, the medication reviews included six follow-up appointments. Many earlier studies investigating CMRs focused on prescribing guidelines and potential for inappropriate prescribing rather than patient goals [4,6,8,10,14,38,39]. Several studies have suggested that patient interviews are important in finding the most relevant DRPs [2,40–42].

In the DREAMeR study, the patient-centred approach of CMR improved outcomes for older patient’s lives such as the EQ-VAS and the number of health problems that affected daily life. Although the EQ-5D health utility values (which this study was powered on) did not change, the more patient-relevant EQ-VAS did change, even though it was a small change of 3.4 points. Taking this in combination with the impact on health problems that affect patients’ lives and a small reduction in the number of medicines used, we consider this of clinical relevance for older patients’ lives. It is a well-known problem that the patient experience is not always adequately represented in the responsiveness of the EQ-5D [43,44]. Therefore, measuring patient-reported outcomes is important to assess the benefits of this type of patient-centred care. Effects of a medication review on specific health problems (such as pain) have been reported earlier [45], but no studies have measured a range of 12 different health problems during medication review in primary care.

Our patient-centred CMR structurally adds patients’ values and preferences to the scientific evidence and the experience of healthcare professionals to complete the three main components of evidence-based medicine. In this study, during the CMRs we specifically focused on patients’ preferences, whereas many previous studies did not structurally involve this aspect [4,6,7]. Shared decision-making involving the GP, pharmacist, and patient needs a stepwise and individualised approach and goal setting [46,47]. In the present study, all these aspects were present during the CMRs. Per the results of our study, goal setting during CMR is important to prioritise the most important problems and motivate the patient for potential interventions, such as a change in drugs.

In this study, both the addition and discontinuation of drugs occurred more frequently in the intervention group. This suggests that, in older patients, a personalised approach can help to balance potential harms and advantages to achieve optimal pharmacotherapy for an individual’s current situation. In this study, the net effect was a slight decrease in the total number of long-term medications, which is noteworthy because it is increasingly accepted that ‘deprescribing’ in older persons with polypharmacy is a major challenge for the coming years [48–50].

Our study has several strengths. First, the novelty of the design was the combination of a complex intervention with personalised goal setting using patient-reported outcomes. Second, proposing personal goals during the medication reviews was new, to our knowledge, and was effective in prioritising the most important problems for patients. Third, this was a pragmatic trial performed in daily pharmacy practice, which might enhance the generalisability of our results. Finally, we included pharmacists with experience in CMR and established cooperation with GPs and stipulated two follow-up contacts with patients by pharmacists and/or GPs, thereby including all the necessary elements for good CMRs [2,40,41].

Some limitations of this trial also need to be addressed. First, because of the nature of the intervention, blinding was not feasible, which might have influenced the results of this trial. To minimise the risk of bias, all questionnaires were captured and recorded by independent research assistants. Patients in the control group were offered a CMR after the end of the 6-month follow-up. Pharmacists are unlikely to have given extra attention to patients in the control group because they would have lacked time to perform additional medication reviews during the study period, but it is possible that these patients could have been prompted to consider obtaining advice about their medication, health problems, or goals by participating in this study. However, this would have led to an underestimation rather than overestimation of the study results. Second, there could be risk of contamination. However, the per protocol analysis showed that there were no differences in results from the intention-to-treat analysis. Third, only 25% of eligible patients participated in this study. We may have missed patients who could have benefited from a CMR (for example, typical healthcare avoiders or persons with language problems or low health literacy). Future studies should think about strategies to specifically reach out to these types of patients. Fourth, there is a risk associated with proposing and measuring goals. For example, when unrealistic or unsolvable goals are proposed by the patient, this can lead to disappointment and a reduction in quality of life. Therefore, it is important to use a SMART formulation for goals. Although we trained pharmacists to formulate realistic goals with a training day and monthly web conferences, we cannot exclude the possibility that some of the goals may not have been realistic. Further education of pharmacists in goal setting is important for future implementation of patient-centred CMRs. Fifth, we did not compare the patient-centred CMRs focused on personal goals with a more traditional CMR. Therefore, it remains difficult to demonstrate which part of this complex intervention (for example, goal setting, extra attention to patients, reducing health problems, other drug changes, etc.) contributed to the effects we observed. However, we are of the opinion that a focus on patient’s preferences during a CMR enables shared decision-making and may motivate the patient to think about their own treatment and to optimise the effects of a CMR. Finally, we only measured quality of life, health problems, and long-term medication use in this study, while the development of a core outcome set (COS) for medication review has been published [25]. However, the DREAMeR study was designed before the introduction of this COS. Nevertheless, most of the mentioned outcomes in this COS are also included in the DREAMeR study, such as pain, quality of life, and DRPs such as over- and underuse or drug–drug interactions [26,37], so most results of our study could be compared with future studies using COS in medication review.

It could be questioned whether this type of CMR could be implemented worldwide. CMRs are performed in different countries, but not always on a structural basis such as that in the Netherlands, because of limitations in remuneration, performance settings, lack of guidelines, and limited effects [1,2,51–54]. As attention to goal-oriented patient care and patient outcomes become increasingly important in the management of multimorbidity and polypharmacy [13,55], implementation of patient-centred CMR is one of the steps that may help to improve older patient’s lives.

## Conclusion

The DREAMeR study was an RCT aiming to assess the effect of a CMR focused on personal goals on quality of life in older persons with polypharmacy in the Netherlands. We observed improvements in quality of life measured by the EQ-VAS, reductions in the number of health problems that patients felt had a moderate to severe impact on daily life, and reductions in the number of prescribed long-term medications, but the CMR had no effect on HR-QoL measured by the EQ-5D-5L or total number of health problems. Including patient’s goals and preferences during the patient interviews of CMR may be important to establish clinically relevant effects of this intervention.

## Supporting information

S1 CONSORT Checklist CONSORT, consolidation standards of reporting trials. (DOC)

S1 Study Protocol (PDF)

S1 Statistical Analysis Plan (DOCX)

S1 Database (PDF)

S1 Table Twelve health problems in questionnaire. (DOCX)

S2 Table Results unadjusted linear mixed model analysis. (DOCX)

S3 Table Participants with problems per domain of the EQ-6D. EQ, EuroQol.
(DOCX)

S4 Table Results per protocol analysis. (DOCX)

S5 Table Top 10 added and ceased drugs. (DOCX)

S6 Table Healthcare consumption. (DOCX)

S7 Table Classification of identified and solved DRPs. DRP, drug-related problem.
(DOCX)

S1 Fig Example health problem ‘pain’ in questionnaire. (DOCX)

S2 Fig Number of drugs over time. (DOCX)

## Abbreviations

ATC: Anatomical Therapeutic Chemical classification
CI: confidence interval
CMR: clinical medication review
CONSORT: Consolidation Standards of Reporting Trials
COS: core outcome set
DREAMeR: Drug use Reconsidered in the Elderly using goal Attainment scales during Medication Review
DRP: drug-related problem
EQ: EuroQol
GAS: goal attainment scaling
GORD: gastro-oesophageal reflux disease
GP: general practitioner
HR-QoL: health-related quality of life
iMTA: Institute for Medical Technology Assessment
IQR: interquartile range
ISCOPE: Integrated Systematic Care for Older People
LDL: low-density lipoprotein
OTC: over the counter
RCT: randomised controlled trial
SD: standard deviation
SMART: specific, measurable, acceptable, realistic, time-bound
VAS: Visual Analogue Scale
