# Supplementary material for: Effects of a clinical medication review focused on personal goals, quality of life, and health problems in older persons with polypharmacy: A randomised controlled trial (DREAMeR-study)
Source: PLoS Med. 2019 May 8;16(5):e1002798. doi: 10.1371/journal.pmed.1002798 (PMC6505828; doi:10.1371/journal.pmed.1002798)
Supplement: S1 Statistical Analysis Plan — (DOCX) [file pmed.1002798.s003.docx]

**Statistical analysis plan – DREAMeR study**

**Objective**

To determine the effect of a CMR focusing on the patient's preferences, health-related complaints and personal goals related to their medication on patients' health-related quality of life and their health-related complaints.

**Primary outcomes**

1. Health-related quality of life
   1. EQ-5D (range
      1. Utilities will be calculated with the aid of EQ-5D tariff
   2. EQ-VAS (range 0-100)
2. Health-related complaints
   1. Number of Health-related complaints independent of severity (range 0-12)
   2. Number of Health-related complaints with an impact on patient’s daily life (range 0-12)

Measurements will be collected through written questionnaires at baseline, T1 (at three months) and T2 (at six months). Six months = endpoint.

**Secondary outcomes**

1. Number of drugs
   1. Mean number of drugs in use per patient
   2. Mean number of drugs added and ceased per patient
      - Measurements are collected through drug dispensing data and the number of drugs in use is calculated during each month.
2. (Healthcare consumption)
   - This outcome measure will only be used for the economic evaluation of the DREAMeR study.
   - Measurements are collected through written telephonic questionnaires of the Medical Consumption Questionnaire at baseline, T1 (at three months) and T2 (at six months).
3. Process outcomes will be measured during the medication reviews, like drug-related problems, interventions, implementation rates of interventions, type of goals and attainment of goals. Most of these results will be extensively described in another paper focused on the intervention group of the DREAMeR study. These results will be presented as means and standard deviations or percentages.

**Sample size calculation**

The sample size is based on an expected change on the EQ-5D utility score of 0.05 ± 0.20 over six months. This difference is considered to be a clinically relevant and feasible, based on previous studies in Spain and the Netherlands [15,26]. This difference indicates a 5% change of HR-QoL measured with EQ-5D, but could also be translated to a 5% change in EQ-VAS. This would indicate an improvement of 5 points on a scale from 0-100 over six months.

To achieve a statistically significant difference in the utility on the EQ-5D with alpha = 0.05 and beta = 0.20, a group size of 252 is sufficient. Allowing for a potential drop-out rate of 25%, a total number of ∼ 630 participants are needed (315 in each group).

This sample size is also expected to be sufficient for the second primary outcome measure: the number of health-related complaints per patient. Because comparative studies with this outcome measure are lacking, we have made some assumptions. If the study population consists of 252 patients per group, a difference on the number of health-related complaints with approximately 0.5±2 with alpha = 0.05 and beta = 0.20 could be demonstrated. We expect that a patient has an average of two health-related complaints with moderate to severe impact on daily life, which may possibly be reduced by 25%. We consider this difference as feasible and clinically relevant. The number of complaints will be highly variable. Therefore we assume a standard deviation of two.

**Statistical analysis**

Descriptive statistics will be used for patient characteristics. Normal distributed values will be described as means and standard deviations and non-normal distributions will be described as means and interquartile ranges or percentages.

Dropout and loss to follow-up will be described. This will be categorised into five different reasons: medical condition, deceased, loss of interest, moved or technical reasons divided per time moment and per group. Differences in drop out between the two groups will be analysed using an independent sample t-test.

Unadjusted scores for the primary outcomes will be reported as means and standard deviations in a table, presented as the means at baseline, T1 and T2 measurement between both groups.

Effect analyses will be performed according to both ‘intention to treat’ and ‘per protocol’ principles. For the per protocol analyses, patients in the control group who did receive a CMR during the study period will be excluded.

Longitudinal differences in the primary outcomes between the two groups will be analysed with linear mixed model analyses, because of repeated measurements (baseline, T1 and T2). Intervention, time (baseline, T1 and T3), and the interaction between intervention and time will be used as fixed factors in the linear mixed model. Participant identification number will be included as a random effect to account for the dependence of repeated observations. The interaction between intervention and time will be the main effect presented as the beta value with the 95% confidence interval. With this beta, the effect at three months and the effect at six months can be calculated separately assuming a linear curve. Baseline characteristics (age, sex and pharmacy) can be integrated into the mixed model to control for confounding, despite of the randomisation, but also a model without adjustment for baseline characteristics will be analysed.

Secondary outcomes will be analysed analogously. For effects on the number of drugs used, a linear mixed model will be performed. The intervention, time (per month) and the interaction between intervention and time will be entered as fixed factors in the model. Participant identification number will be included as a random effect to account for the dependence of repeated observations. The number of drugs added and ceased will be calculated based on the drug dispensing data, extracting the number of drugs per patient at baseline from the number of drugs used per patient at six months. An independent samples t-test will calculated the difference in the average number of drugs added and ceased per patient at six months between both groups.

Per type of health-related complaint, the percentage of patients with complaints will be presented in a table split into control or intervention group. Mean severity scores (+ standard deviation) per complaint at baseline will be presented. To calculate the differences in severity per complaint (expressed as VAS-score with a range from 0-10) over time between both groups, linear mixed model analyses will be performed, because of repeated measurements. Intervention, time (baseline, T1 and T2), and the interaction between intervention and time will be used as fixed factors in the linear mixed model. Participant identification number will be included as a random effect to account for the dependence of repeated observations. The interaction between intervention and time will be the main effect presented as the beta value with the 95% confidence interval. With this beta, the effect at three months and the effect at six months can be calculated separately assuming a linear curve.

In case of missing data, sensitivity analyses will be conducted to examine the influence of missing data on the study findings. No imputation will be performed for missing data in the primary outcomes, because a linear mixed model takes missing values into account. Missing values will be encoded as “999” in the dataset.

Data will be analysed using IBM SPSS Statistics 24.0 (IBM Corporation, Armonk, NY, USA).

A p-value of ≤ 0.05 will be considered significant.
