# Supplementary material for: Effects of a clinical medication review focused on personal goals, quality of life, and health problems in older persons with polypharmacy: A randomised controlled trial (DREAMeR-study)
Source: PLoS Med. 2019 May 8;16(5):e1002798. doi: 10.1371/journal.pmed.1002798 (PMC6505828; doi:10.1371/journal.pmed.1002798)
Supplement: S1 Database — (PDF) [file pmed.1002798.s004.pdf]

ID	Group	Pharmacy	Age	Gender	Utility_t0	EQ-VAS_T0	Utility_T1	EQ-VAS_T1	Utility_T2	EQ-VAS_T2	Total_health_problem	Total_health_problem	Total_health_problem	Health_problems_with_impact	Health_problems_with_impact	Health_problems_with_impact_T2
1	1	1	84	1	0,781	70	0,781	70	0,798	70	6	6	4	3	2	1
2	0	1	78	1	0,788	95	0,813	95	999	999	3	3	999	0	1	999
3	1	1	84	1	0,824	50	0,807	60	0,775	60	6	4	5	2	4	1
4	0	1	85	0	1	63	1	80	1	70	5	4	3	2	0	0
5	1	1	89	1	0,798	80	0,7	75	0,776	85	6	8	4	3	4	1
6	0	1	76	0	1	100	999	999	999	999	1	999	999	0	999	999
7	1	1	75	0	0,912	70	0,912	75	0,893	85	5	3	4	1	1	1
8	0	1	79	1	0,893	90	0,912	90	0,893	100	1	2	1	1	1	1
9	1	1	83	1	0,843	80	0,874	100	0,833	100	1	2	0	0	0	0
10	1	1	75	0	0,799	50	1	60	1	85	6	1	3	4	0	0
11	0	1	71	1	0,584	60	0,432	60	0,775	75	6	9	4	6	5	2
12	1	1	89	1	0,707	40	0,657	40	999	999	7	999	6	4	999	5
13	0	1	80	0	0,807	45	0,845	30	0,807	60	9	7	7	9	6	7
14	1	1	79	1	0,805	70	0,798	80	0,792	60	7	8	7	6	0	3
15	0	1	78	0	1	85	0,917	65	0,73	40	0	0	1	0	0	0
16	1	1	92	1	0,385	65	0,575	65	0,374	60	9	10	10	6	6	5
17	0	1	75	0	0,396	20	999	999	999	999	10	999	999	8	999	999
18	1	1	89	0	0,867	75	0,798	80	0,798	70	4	5	5	2	2	1
19	0	1	82	0	0,824	75	0,824	80	0,824	75	4	2	2	4	2	2
20	1	1	81	0	0,867	70	0,835	60	0,756	50	7	12	3	4	3	3
21	0	1	81	1	1	80	1	80	1	80	0	3	0	0	0	0
22	1	1	79	0	0,776	100	0,632	50	0,75	90	6	6	4	2	6	1
23	0	1	80	0	0,912	85	0,912	86	0,912	80	4	3	5	0	3	0
24	0	2	71	1	0,651	70	0,464	40	999	999	10	8	999	8	7	999
25	0	2	73	1	1	95	1	90	1	85	0	0	0	0	0	0
26	0	2	78	1	0,843	90	0,843	85	999	999	5	5	999	3	4	999
27	0	2	82	1	0,807	85	0,497	50	0,67	40	5	5	6	2	2	5
28	0	2	78	1	0,707	75	0,837	70	0,805	55	7	8	11	4	4	4
29	0	2	89	1	0,322	25	0,058	20	0,067	10	9	11	9	6	4	7
30	0	2	73	1	0,003	50	0,231	70	0,606	70	10	10	9	9	6	9
31	0	2	72	1	0,788	75	0,805	80	0,805	80	4	4	3	2	3	0
32	0	2	82	1	0,454	60	0,684	85	0,394	55	9	5	7	9	4	5
33	0	2	78	1	0,788	90	0,876	80	0,861	75	2	2	4	1	0	0
34	0	2	76	0	0,503	80	999	70	0,49	80	3	3	3	3	3	3
35	0	2	81	1	0,584	60	0,781	50	999	999	5	8	999	5	6	999
36	0	2	80	0	0,917	70	0,917	70	0,917	75	5	5	4	1	1	0
37	0	2	78	0	0,912	85	0,912	95	0,867	70	3	3	3	0	3	3
38	1	3	81	1	0,845	70	999	999	999	999	6	999	999	2	999	999
39	1	3	83	1	0,912	95	1	90	0,833	80	4	4	5	1	1	0
40	0	3	81	1	0,874	75	0,733	65	0,745	60	4	6	4	0	1	1
41	1	3	85	1	0,467	75	0,416	40	999	999	8	9	999	6	6	999
42	0	3	87	0	0,73	70	0,681	65	0,699	80	12	10	4	8	4	3
43	1	3	78	1	0,683	75	0,651	45	0,677	55	11	12	11	6	7	7
44	0	3	79	1	0,713	75	0,418	40	0,179	50	7	5	9	4	1	5
45	1	3	72	0	0,833	70	0,781	65	0,805	75	8	10	9	0	3	2
46	0	3	71	1	1	90	0,833	75	1	80	5	7	5	5	5	1
47	1	3	86	1	0,7	75	0,7	75	0,7	70	5	5	4	4	2	2
48	0	3	84	0	0,683	75	0,813	70	0,623	30	0	6	6	0	5	6
49	1	3	81	1	0,781	50	0,781	65	0,775	65	5	5	4	1	1	2
50	0	3	72	0	0,874	90	0,874	90	0,874	80	4	5	3	0	0	0
51	1	3	83	0	0,833	80	0,806	85	0,817	80	2	4	2	1	3	0
52	0	3	90	1	0,569	45	0,483	999	0,483	45	8	7	6	6	2	3
53	1	3	79	0	0,781	70	0,824	70	0,798	60	9	5	3	1	1	1
54	0	3	78	1	0,693	70	0,693	65	0,693	55	3	3	3	3	2	2
55	1	3	84	0	0,793	80	0,763	85	0,713	80	7	4	8	4	0	7
56	0	3	80	1	1	60	0,781	50	0,664	55	1	2	7	0	2	5
57	1	3	82	1	0,444	80	0,285	50	0,651	60	6	5	5	5	3	5
58	1	4	86	1	0,749	60	0,719	60	0,805	50	8	6	5	2	4	2
59	0	4	88	1	0,756	75	0,833	90	1	999	4	2	1	4	0	0
60	1	4	77	0	0,876	75	0,798	85	0,798	80	6	5	6	0	0	0
61	0	4	73	1	0,775	75	0,835	70	0,781	75	7	8	8	6	7	7
62	1	4	91	1	0,281	70	0,514	60	0,67	60	7	7	6	3	6	3
63	1	4	78	0	0,749	50	0,775	80	0,391	70	9	7	6	5	5	6
64	0	4	81	1	0,657	999	0,683	60	999	999	7	999	11	6	999	5
65	1	4	77	0	0,874	80	0,737	80	0,765	80	6	5	6	0	0	0
66	0	4	77	0	0,912	100	0,912	100	0,833	95	1	4	2	0	0	0
67	1	4	86	1	0,378	30	0,448	70	0,176	75	5	5	6	5	5	5
68	0	4	81	0	0,874	75	0,689	60	999	999	3	3	999	0	3	999
69	1	4	78	0	0,775	50	0,792	60	0,7	60	11	11	6	5	4	5
70	0	4	73	0	0,615	50	0,788	65	0,781	70	7	6	8	6	1	4
71	1	4	77	1	0,139	22	0,348	40	0,405	50	9	8	9	9	6	6
72	0	4	77	0	0,664	20	0,71	60	0,675	35	10	7	6	6	5	6
73	1	4	82	0	0,689	50	0,621	70	0,638	65	12	6	8	7	2	3
74	0	4	82	0	0,912	85	0,833	80	1	85	1	2	2	0	0	0

Legend

Group	0	control
	1	intervention
Seks	0	male
	1	female
Missing	999	missing value

75	1	4	83	1	1	80	0,833	90	0,912	80	2	3	1	0	0	0
76	0	4	78	1	0,693	45	0,707	75	0,693	50	3	5	4	3	4	3
77	1	4	79	1	1	80	0,874	80	0,874	75	3	4	4	0	0	0
78	1	4	89	0	0,7	80	0,867	70	0,893	80	2	4	1	1	3	1
79	1	4	80	0	0,651	65	0,651	65	0,583	65	5	5	4	3	5	4
80	0	4	82	1	0,874	80	0,837	80	0,874	75	5	3	3	2	0	2
81	1	4	72	0	0,807	75	0,798	75	999	999	2	2	999	1	1	999
82	0	4	84	1	0,497	70	0,755	70	0,781	55	4	4	3	4	2	2
83	0	5	71	1	0,621	70	0,595	50	999	999	5	7	999	4	5	999
84	1	5	84	1	0,775	50	0,73	70	999	999	8	8	999	5	3	999
85	1	6	76	0	0,781	60	0,664	80	999	70	3	4	8	1	3	7
86	0	6	74	0	1	75	1	80	1	80	1	0	0	0	0	0
87	1	6	82	1	0,707	70	0,707	80	0,399	90	10	10	9	8	8	7
88	0	6	80	1	0,454	55	0,805	90	0,715	75	4	6	7	3	1	1
89	1	6	89	0	0,765	85	0,912	85	0,765	85	2	5	5	0	1	0
90	0	6	76	0	0,678	50	0,501	50	0,681	50	6	8	5	5	5	2
91	1	6	84	0	0,874	85	0,874	90	0,843	85	5	6	5	0	0	0
92	0	6	78	0	0,7	75	0,733	70	0,707	80	7	6	8	1	0	0
93	1	6	84	1	0,565	45	0,775	65	0,591	90	8	8	7	7	7	6
94	0	6	88	1	0,755	75	0,687	70	0,704	60	5	4	5	3	1	2
95	1	6	71	1	0,845	95	0,845	85	0,845	90	2	3	3	0	0	0
96	0	6	73	1	0,798	75	0,805	80	0,805	75	4	4	4	2	4	3
97	1	6	79	0	0,874	80	1	85	1	80	3	4	5	0	0	0
98	0	6	71	1	0,799	50	0,788	65	0,781	50	5	5	7	3	4	5
99	1	6	76	0	0,833	80	0,813	85	0,805	90	3	4	3	3	2	0
100	0	6	78	1	0,589	50	0,657	60	0,677	60	10	9	6	4	4	3
101	1	6	81	1	0,807	70	0,755	90	0,824	95	4	3	3	2	2	0
102	0	6	75	0	0,837	70	0,874	75	999	999	5	3	999	0	0	999
103	0	7	76	1	0,597	70	0,707	65	999	999	6	6	999	3	3	999
104	1	7	73	0	0,394	55	-0,012	30	999	999	12	12	999	9	5	999
105	0	7	80	1	0,385	70	0,499	70	999	999	5	5	999	4	3	999
106	1	7	75	0	0,689	65	0,874	50	999	999	9	8	999	6	7	999
107	0	7	85	1	0,348	50	0,084	30	999	999	12	10	999	3	6	999
108	1	7	79	0	0,674	70	999	999	999	999	10	999	999	7	999	999
109	0	7	74	0	0,781	90	0,817	90	0,788	90	3	2	3	2	1	1
110	1	7	80	0	0,912	75	0,798	80	0,798	80	4	10	8	0	0	0
111	1	7	75	0	0,775	50	0,833	75	0,833	85	7	4	4	2	0	0
112	0	7	81	1	0,912	80	0,912	80	999	999	1	999	1	1	999	1
113	0	7	85	0	0,874	55	0,874	70	999	999	8	999	6	1	999	1
114	1	7	77	0	0,612	50	0,612	60	0,687	60	7	4	4	5	1	3
115	1	7	81	1	0,71	50	999	999	999	999	4	999	999	2	999	999
116	0	7	81	0	1	70	0,837	70	0,707	70	3	3	7	1	2	2
117	1	7	81	1	0,775	60	0,707	50	0,584	50	10	9	9	8	9	4
118	0	7	81	0	0,385	55	0,359	50	0,334	50	9	8	8	6	4	4
119	0	7	82	0	0,824	80	0,912	80	0,912	80	7	6	10	2	4	4
120	0	8	71	1	0,845	80	0,715	70	0,765	75	1	4	2	0	0	0
121	1	8	73	1	0,713	80	0,608	65	0,713	60	10	11	10	7	9	7
122	0	8	74	0	1	70	1	85	1	90	1	1	1	1	1	1
123	1	8	81	0	0,912	60	0,867	50	0,088	999	8	7	5	2	2	5
124	0	8	77	0	0,781	80	0,7	50	999	65	4	3	6	3	3	2
125	1	8	77	0	0,867	80	0,841	85	0,867	90	2	1	1	1	1	1
126	0	8	71	0	0,833	90	0,824	85	999	999	1	3	999	1	1	999
127	1	8	90	1	0,837	99	0,805	70	0,837	85	7	7	9	2	1	1
128	0	8	85	1	0,7	80	0,707	80	0,366	70	2	3	4	0	0	0
129	1	8	79	1	0,739	60	0,775	70	0,781	80	4	6	6	4	4	6
130	0	8	71	0	0,805	85	0,805	80	0,805	80	6	6	7	0	0	0
131	1	8	83	1	0,529	50	999	75	999	80	10	9	5	7	7	3
132	0	8	87	0	0,643	75	0,535	40	0,364	75	3	9	7	2	4	7
133	1	8	79	0	0,756	999	999	999	999	999	4	999	999	0	999	999
134	1	8	81	0	0,833	70	0,798	70	0,798	70	5	6	5	0	2	4
135	0	8	82	1	0,31	60	999	999	999	999	8	999	999	2	999	999
136	1	8	71	1	0,486	55	0,788	40	0,775	65	6	6	4	6	4	3
137	0	8	81	0	0,792	80	0,867	80	0,724	80	6	3	4	2	1	2
138	1	8	72	0	0,775	70	0,813	85	0,7	80	9	9	10	2	2	2
139	1	8	84	1	999	75	0,805	95	0,713	50	3	6	7	0	0	4
140	0	8	70	0	0,805	75	0,733	70	1	75	9	6	7	2	2	1
141	1	8	79	1	0,912	70	0,799	80	0,798	70	9	11	7	2	0	1
142	0	8	87	1	0,274	60	999	999	999	999	4	999	999	3	999	999
143	1	8	80	1	0,805	90	0,833	90	0,798	95	3	5	6	0	2	1
144	0	8	86	0	0,621	75	0,601	60	0,707	70	6	4	9	1	1	3
145	1	9	74	0	0,329	40	0,765	70	0,474	50	5	3	6	5	0	1
146	0	9	85	0	1	90	1	80	1	85	2	2	2	0	0	0
147	1	9	86	1	0,049	50	0,807	999	0,355	50	7	4	3	4	3	2
148	0	9	83	1	0,522	75	0,677	80	0,689	75	2	4	4	1	2	0
149	1	9	85	0	0,503	70	0,408	43	0,394	12	6	6	6	2	5	5

150	0	9	77	1	0,689	55	0,707	55	0,677	70	5	5	6	4	5	6
151	1	9	74	0	1	70	0,874	88	0,874	90	3	5	3	0	0	0
152	0	9	76	1	0,788	80	0,781	70	0,7	55	6	5	6	3	3	2
153	1	9	79	0	1	83	1	85	1	85	0	0	0	0	0	0
154	0	9	75	0	0,73	60	0,781	65	0,805	65	7	7	10	2	1	2
155	1	9	83	0	0,707	50	0,615	50	0,724	60	12	9	9	6	2	5
156	0	9	74	0	0,807	90	0,775	60	0,792	70	2	6	5	1	2	0
157	1	9	82	0	0,912	75	0,833	75	0,893	70	2	6	4	0	0	1
158	0	9	83	1	0,775	90	0,775	85	0,775	80	4	4	4	4	4	4
159	1	9	81	0	0,7	75	0,813	75	0,833	80	9	10	7	1	0	1
160	0	9	83	0	0,694	50	0,805	60	0,625	60	6	4	6	1	1	2
161	1	9	97	1	0,677	60	0,681	80	999	999	10	12	999	9	9	999
162	0	9	80	1	0,781	45	0,392	45	-0,002	50	8	6	5	4	5	5
163	1	9	77	1	0,833	80	0,833	95	0,833	95	8	2	2	0	0	0
164	0	9	77	1	0,745	60	0,843	70	0,833	75	2	4	5	1	3	0
165	1	9	85	1	0,516	70	0,73	65	0,833	75	8	7	7	4	5	2
166	0	9	84	1	0,664	70	0,707	70	0,776	60	6	5	4	5	3	2
167	1	9	82	1	0,621	60	0,327	20	999	999	3	3	999	2	3	999
168	0	9	73	1	0,737	75	999	999	999	999	5	999	999	0	999	999
169	1	9	82	0	1	80	1	80	1	80	0	1	0	0	0	0
170	0	9	81	1	0,775	75	0,565	80	0,775	70	7	7	7	7	6	6
171	1	9	80	1	0,805	65	0,799	60	0,805	65	5	5	5	3	3	1
172	0	9	77	1	0,833	80	0,833	80	0,833	80	4	6	9	0	0	1
173	1	10	89	1	0,707	75	0,7	75	999	999	12	999	9	5	999	4
174	0	10	80	1	0,833	80	0,805	80	0,805	75	6	8	7	1	2	2
175	1	10	75	0	1	90	1	90	1	90	1	3	1	0	0	1
176	0	10	90	1	0,458	65	0,292	65	0,619	55	9	8	9	4	6	8
177	1	10	91	1	0,867	90	999	90	0,798	80	4	5	5	2	3	3
178	0	10	71	0	0,296	40	0,586	50	0,464	20	9	4	6	6	2	4
179	1	10	72	0	0,769	90	999	999	999	999	0	0	0	0	0	0
180	0	10	82	0	0,781	55	0,709	55	0,709	70	6	6	8	3	4	7
181	1	10	79	0	0,446	50	0,608	60	0,677	50	2	3	4	2	3	3
182	0	10	84	1	0,281	70	0,213	60	0,342	65	9	5	5	6	5	5
183	1	10	73	0	1	90	1	90	999	999	1	0	999	0	0	999
184	0	10	72	0	0,912	70	0,912	80	0,824	70	3	4	4	0	0	0
185	0	10	79	0	0,709	60	0,709	70	0,683	55	6	7	7	3	3	3
186	1	10	76	0	0,804	75	0,683	70	0,733	70	7	7	8	5	6	4
187	1	10	80	1	0,615	70	0,798	80	999	999	3	2	999	3	0	999
188	0	10	86	0	0,73	60	0,606	60	999	999	6	8	999	2	4	999
189	0	10	76	1	0,724	50	0,792	65	0,606	60	5	6	6	3	4	1
190	1	10	94	0	0,698	70	0,684	75	0,588	70	5	4	6	2	3	2
191	0	10	100	1	0,266	65	0,353	57	999	999	11	999	9	10	999	4
192	1	10	84	1	0,824	75	999	85	999	999	5	2	999	0	0	999
193	0	10	76	0	1	75	1	80	0,874	80	4	2	3	0	0	0
194	1	10	88	1	0,448	60	999	999	999	999	8	999	999	5	999	999
195	1	10	79	1	0,713	70	0,608	60	0,713	65	7	7	6	5	4	3
196	1	10	86	1	0,53	60	0,775	50	0,799	50	6	6	3	3	4	0
197	0	10	79	1	0,874	80	999	999	0,874	80	6	3	5	0	0	1
198	1	10	87	0	0,798	80	0,833	90	0,807	95	4	3	5	4	2	5
199	1	11	83	1	0,745	75	0,569	65	0,699	65	8	9	10	8	5	5
200	1	11	75	0	1	80	1	95	1	95	6	3	3	0	1	0
201	0	11	76	1	0,182	50	0,182	50	0,67	70	6	7	8	3	3	3
202	1	11	72	0	0,707	80	999	50	0,737	80	6	4	4	2	0	0
203	0	11	70	1	0,597	50	999	999	999	999	7	999	999	3	999	999
204	1	11	70	1	0,805	70	0,733	75	999	999	6	6	999	0	2	999
205	0	11	72	0	0,824	80	0,833	90	0,833	95	5	4	4	0	0	0
206	1	11	72	1	0,912	80	0,833	90	0,833	90	7	5	6	0	0	0
207	0	11	71	0	0,687	80	0,749	60	0,416	80	4	4	4	3	4	4
208	1	11	81	0	0,837	70	0,912	70	0,805	85	1	3	5	0	3	1
209	1	11	80	1	0,677	60	0,775	70	999	999	4	4	999	2	1	999
210	1	11	76	0	0,769	80	0,798	80	0,769	80	5	4	1	2	2	0
211	1	12	72	1	0,399	60	0,46	50	0,687	45	9	8	8	5	5	5
212	1	12	76	1	0,775	80	0,29	20	0,49	50	6	9	3	1	3	0
213	1	12	78	0	0,843	75	0,843	80	0,813	80	1	2	3	0	0	0
214	0	12	83	0	0,833	75	0,781	70	0,719	80	5	4	4	4	4	3
215	1	12	82	1	999	70	0,837	80	0,917	80	2	2	1	1	0	0
216	0	12	72	0	0,835	80	0,707	70	0,698	70	4	4	4	2	1	2
217	1	12	83	0	0,798	60	0,719	60	999	999	7	6	999	4	6	999
218	0	12	73	0	0,776	70	0,484	80	0,588	30	3	3	3	3	3	3
219	0	12	86	1	0,719	80	0,73	60	0,638	60	7	7	7	2	3	3
220	1	12	81	0	0,833	85	999	999	999	999	5	999	999	1	999	999
221	1	12	81	1	0,73	80	0,707	70	0,707	85	12	8	8	4	2	2
222	0	12	88	0	0,687	70	0,608	75	0,681	65	5	7	6	2	4	4
223	1	12	77	0	0,7	70	0,683	60	999	999	8	999	11	0	999	0
224	0	12	74	0	0,837	80	0,917	85	1	80	4	3	2	0	2	1

225	1	12	77	1	0,625	55	0,805	55	0,792	65	10	9	6	5	2	4
226	0	12	86	1	0,813	85	0,833	90	0,912	95	4	3	1	0	0	0
227	1	12	82	1	0,874	90	0,805	65	0,837	60	4	2	2	0	2	0
228	0	12	81	0	0,73	80	0,833	75	0,733	70	10	4	3	1	1	3
229	1	12	78	1	0,781	70	0,781	70	0,781	70	8	5	7	3	2	3
230	1	13	75	1	0,213	35	0,399	45	0,446	60	5	4	2	3	3	2
231	0	13	71	1	0,845	95	0,845	95	0,845	95	1	2	1	1	1	1
232	1	13	71	1	0,677	70	0,73	70	0,781	60	8	2	6	3	1	2
233	0	13	71	1	0,683	60	0,745	75	0,713	75	4	4	4	1	2	3
234	1	13	83	0	0,912	90	0,805	65	0,876	75	3	5	6	1	0	1
235	0	13	76	0	0,359	50	0,667	30	0,588	50	6	7	6	6	6	5
236	1	13	83	1	0,588	40	0,707	65	0,805	70	10	8	6	9	5	3
237	0	13	73	1	0,707	70	0,713	60	0,707	60	7	7	5	5	4	2
238	1	13	77	1	0,129	30	0,16	25	0,144	60	4	5	3	3	5	3
239	0	13	80	1	0,591	85	0,775	75	0,677	70	5	4	7	2	2	7
240	1	13	83	1	0,683	60	0,683	50	0,689	75	11	7	7	3	2	3
241	0	13	86	1	0,745	70	0,73	70	0,813	70	8	3	4	4	3	4
242	1	13	76	0	0,912	80	0,912	80	0,837	80	5	7	6	2	1	0
243	0	13	79	1	0,813	40	0,874	75	999	999	9	11	999	8	11	999
244	1	13	79	0	0,707	75	0,833	85	0,737	90	7	4	5	2	1	2
245	0	13	81	1	0,833	80	0,813	80	0,833	80	6	8	6	0	2	0
246	1	13	81	1	0,805	60	0,874	50	0,837	80	4	9	5	0	1	0
247	0	13	87	1	0,677	60	0,677	65	0,694	65	12	12	11	5	7	5
248	1	13	83	1	0,588	45	0,432	55	0,432	50	11	9	12	7	5	6
249	0	13	80	0	0,833	90	0,707	85	0,912	95	3	3	2	0	0	0
250	1	13	82	1	0,874	95	1	95	0,912	80	2	1	2	0	0	0
251	0	13	80	1	0,208	50	0,502	55	0,394	50	9	7	8	7	6	6
252	1	13	77	1	0,775	90	0,775	80	0,749	60	6	4	7	4	2	5
253	1	13	82	1	0,781	75	0,781	70	0,781	70	9	9	6	2	4	2
254	1	13	71	1	0,737	60	0,765	999	1	85	3	1	0	2	0	0
255	1	14	83	0	0,897	70	1	70	1	80	5	4	3	1	1	1
256	1	14	89	1	0,794	70	1	75	1	75	4	2	4	1	2	3
257	0	14	73	0	0,833	90	0,805	85	0,833	95	4	4	2	0	0	0
258	0	14	89	1	0,837	80	0,843	80	0,874	80	3	4	3	0	0	0
259	0	14	85	1	1	75	1	80	0,876	90	2	2	5	0	0	1
260	1	14	82	0	1	80	0,912	85	1	94	1	3	0	0	0	0
261	0	14	72	1	0,719	50	999	999	999	999	2	999	999	1	999	999
262	1	14	88	0	0,837	90	0,874	90	0,874	90	4	2	3	0	1	0
263	0	14	79	1	0,557	55	0,677	50	0,677	60	8	7	7	6	6	7
264	1	14	74	1	0,584	45	0,419	50	0,54	80	5	5	7	2	1	0
265	0	14	77	0	0,652	55	0,824	70	0,874	60	5	4	3	5	2	1
266	1	14	75	0	0,874	75	0,912	75	999	999	6	1	999	0	0	999
267	0	14	89	0	0,808	80	0,805	80	0,75	65	7	7	10	1	1	1
268	1	14	73	1	0,798	60	0,798	10	0,749	35	5	2	5	1	1	1
269	0	14	84	1	0,835	80	0,518	85	0,773	75	2	2	2	1	2	1
270	1	14	83	1	0,805	80	0,707	60	0,798	50	6	3	12	0	2	5
271	1	15	77	0	0,696	70	999	999	999	999	5	999	999	4	999	999
272	0	15	84	1	0,788	90	999	95	0,805	75	7	6	7	4	3	0
273	1	15	74	1	0,438	50	0,608	55	999	999	6	6	999	6	5	999
274	0	15	88	1	999	40	0,781	30	0,775	70	10	11	12	3	9	7
275	1	15	73	1	0,874	75	0,874	75	0,833	90	2	1	1	0	0	0
276	1	15	78	1	0,683	60	0,683	60	0,683	60	10	10	9	3	6	6
277	0	15	85	1	0,677	55	0,677	50	0,322	40	8	6	8	8	3	4
278	1	15	82	1	0,745	45	0,566	50	0,657	75	10	7	6	5	4	3
279	0	15	86	1	0,781	25	999	75	0,799	75	4	3	2	0	0	0
280	1	15	73	1	0,569	45	0,595	40	0,411	35	12	8	12	4	5	6
281	1	16	77	0	0,799	30	999	999	999	999	11	999	999	4	999	999
282	0	16	72	1	1	80	1	80	1	80	1	1	1	0	0	0
283	1	16	78	1	0,595	50	999	70	0,775	60	10	11	9	6	5	7
284	0	16	77	1	0,608	75	0,775	65	0,775	70	12	9	10	9	6	6
285	1	16	81	1	0,677	65	0,683	999	0,657	50	9	8	7	5	4	5
286	0	16	82	0	0,73	35	0,781	45	0,737	45	10	9	11	3	2	3
287	1	16	76	1	0,805	70	0,805	90	0,805	80	4	5	2	0	0	0
288	0	16	83	0	0,805	80	0,805	95	0,525	40	3	8	6	1	2	3
289	1	16	77	1	0,806	50	0,833	80	0,876	50	5	4	6	5	4	4
290	0	16	72	0	0,733	80	0,765	70	0,843	70	2	1	2	0	0	0
291	1	16	80	0	0,807	65	0,798	75	0,713	70	4	4	4	3	1	2
292	1	16	85	1	0,775	60	999	65	0,565	55	7	8	5	5	6	5
293	0	16	72	0	0,694	55	0,792	60	0,694	55	4	4	5	1	1	1
294	1	16	72	0	1	40	999	999	999	999	0	999	999	0	999	999
295	0	16	73	0	0,165	50	0,373	40	999	999	11	11	999	6	6	999
296	1	16	77	1	0,441	40	0,348	30	0,46	85	6	5	12	5	5	11
297	0	16	85	0	0,799	80	0,7	80	0,792	80	5	4	4	3	3	0
298	1	16	79	1	0,657	50	0,1	50	-0,058	35	8	10	10	7	9	8
299	0	16	87	0	0,597	75	0,773	65	0,704	60	12	12	5	6	3	1

300	1	16	82	1	0,874	999	0,837	80	0,657	80	7	9	8	1	3	5
301	0	16	74	0	0,798	70	0,737	70	0,805	80	7	8	7	0	0	0
302	0	16	81	1	0,833	80	0,833	85	0,805	75	7	8	6	1	1	1
303	1	16	83	1	0,707	45	999	43	0,693	40	5	5	6	1	4	3
304	0	16	93	1	0,833	80	0,805	50	0,805	70	7	5	1	0	1	0
305	1	17	74	0	0,795	60	0,807	60	0,795	60	9	4	2	5	2	1
306	0	17	74	1	0,589	65	0,687	78	0,589	45	11	6	8	4	2	0
307	1	17	84	1	0,707	50	0,668	55	0,808	60	3	2	2	0	0	0
308	0	17	75	1	0,833	80	0,798	79	0,833	80	2	1	2	0	0	0
309	1	17	77	1	0,681	70	0,749	70	0,681	50	5	5	8	3	5	6
310	0	17	89	0	0,612	50	999	999	999	999	10	999	999	5	999	999
311	1	17	93	1	0,638	65	0,583	50	0,632	45	9	6	5	4	3	4
312	0	17	83	1	0,608	70	0,615	70	0,608	70	10	10	9	7	8	9
313	1	17	76	0	0,874	95	0,874	99	0,874	95	1	1	1	0	0	0
314	0	17	79	1	0,805	80	999	75	999	999	7	7	999	0	0	999
315	1	17	83	1	0,622	50	0,51	75	999	35	10	9	10	6	5	3
316	1	17	83	0	1	90	1	90	0,874	80	5	5	8	0	0	0
317	0	17	73	1	0,707	50	0,775	30	999	999	8	6	999	5	2	999
318	1	17	89	1	0,552	80	999	999	999	999	6	999	999	4	999	999
319	0	17	75	0	0,503	60	0,781	30	999	999	8	6	999	5	5	999
320	1	17	75	1	0,683	70	0,486	55	0,683	55	7	6	6	4	3	4
321	0	17	80	1	0,638	60	0,632	65	999	999	4	5	999	4	4	999
322	1	17	87	1	0,083	30	-0,143	20	-0,213	10	8	11	10	8	7	10
323	0	17	82	1	0,689	75	0,689	70	999	999	7	7	999	0	1	999
324	1	17	90	1	0,792	80	0,779	70	0,527	60	8	8	10	4	7	8
325	0	17	81	1	0,775	80	0,713	70	999	999	9	7	999	2	1	999
326	0	17	85	1	0,385	20	0,565	45	999	999	6	6	999	4	3	999
327	1	17	80	1	0,597	80	0,874	90	0,833	95	7	1	5	2	0	0
328	0	17	93	0	0,781	75	999	999	999	999	8	999	999	1	999	999
329	1	17	88	0	0,843	55	999	999	999	999	3	999	999	1	999	999
330	0	17	85	1	0,399	30	999	999	999	999	8	999	999	5	999	999
331	1	17	79	1	0,418	60	0,405	65	0,486	55	6	9	12	4	7	7
332	1	18	71	0	0,837	85	0,876	80	0,917	80	4	4	5	2	2	0
333	0	18	75	1	0,805	80	0,833	75	0,837	75	10	9	10	7	0	5
334	1	18	80	0	0,781	25	0,616	40	0,707	25	10	10	10	6	6	8
335	0	18	85	1	0,798	75	0,805	70	0,775	80	5	8	5	3	2	2
336	1	18	80	0	0,595	60	0,693	60	999	999	6	6	999	6	6	999
337	0	18	83	1	0,807	55	0,813	50	0,788	50	7	6	9	3	4	5
338	0	18	75	1	0,249	50	0,416	30	0,18	40	9	8	9	4	7	5
339	1	18	79	1	0,773	50	0,753	50	999	999	2	999	4	2	999	4
340	0	18	74	0	0,657	50	0,733	40	0,674	80	6	5	8	3	1	4
341	1	18	83	0	0,614	75	0,775	75	0,792	75	5	2	3	5	1	1
342	0	18	71	1	0,516	40	0,483	80	999	999	11	9	999	9	7	999
343	1	18	76	0	0,788	85	0,781	85	0,874	95	9	11	8	2	1	0
344	0	18	89	0	0,893	85	0,912	90	0,912	95	2	2	2	1	0	0
345	1	18	92	1	0,687	60	999	999	999	999	6	999	999	5	999	999
346	0	18	74	0	1	85	0,843	80	1	90	5	7	3	4	3	2
347	0	18	76	0	0,807	70	999	999	999	999	2	999	999	0	999	999
348	0	18	83	0	0,707	70	999	70	0,664	70	8	10	10	2	6	7
349	1	18	75	1	0,7	60	999	999	999	999	6	999	999	1	999	999
350	0	18	72	1	0,824	45	0,833	40	999	999	11	999	6	3	999	3
351	1	18	87	1	0,458	30	999	999	999	999	6	999	999	4	999	999
352	0	18	77	0	0,709	90	0,518	75	999	999	10	999	9	10	999	10
353	1	18	79	1	0,798	75	0,639	70	999	999	6	999	7	4	999	4
354	1	18	79	1	0,486	55	0,713	55	0,122	60	9	7	10	7	5	2
355	0	18	78	1	0,46	80	0,657	50	0,613	70	8	6	10	6	1	3
356	0	18	82	1	0,837	70	0,817	60	0,837	85	1	3	3	0	3	0
357	1	18	84	0	0,645	50	0,799	60	999	999	6	5	999	6	3	999
358	1	19	77	0	0,27	20	0,334	30	0,296	30	10	10	9	7	6	6
359	0	19	77	1	0,713	70	0,805	75	0,615	75	7	7	5	6	3	3
360	1	19	76	0	0,912	75	0,833	70	0,874	80	2	2	5	0	1	2
361	1	19	86	1	0,595	70	0,725	70	0,867	95	8	6	5	6	5	2
362	0	19	71	0	0,399	50	0,687	55	0,773	55	4	4	4	2	1	0
363	1	19	71	1	0,565	60	0,565	30	0,749	55	10	8	8	7	6	5
364	0	19	71	1	0,805	95	0,788	80	0,788	75	8	6	6	1	4	3
365	1	19	86	1	0,509	50	0,502	60	0,56	50	8	8	7	3	4	2
366	0	19	90	0	0,793	50	0,724	65	0,724	60	8	9	8	6	7	2
367	1	19	80	0	0,798	75	0,824	60	0,893	80	3	1	1	0	1	0
368	0	19	76	1	0,622	60	0,448	50	0,707	50	11	10	12	5	7	5
369	1	19	73	0	1	85	1	80	1	90	1	0	0	0	0	0
370	0	19	82	1	0,464	65	0,535	70	0,394	30	12	7	11	4	5	3
371	1	19	72	0	0,912	80	1	85	0,733	85	5	1	5	1	1	0
372	0	19	76	1	0,575	75	0,723	80	0,601	70	5	5	4	5	5	1
373	1	19	72	1	0,583	60	0,608	70	0,792	60	10	10	9	5	5	7
374	0	19	82	0	0,707	60	0,615	55	0,464	45	8	7	10	2	5	2

375	1	19	72	0	0,502	70	0,664	65	0,601	60	8	6	6	6	6	5
376	0	19	74	0	0,737	80	0,737	75	0,707	55	8	6	10	0	0	8
377	1	19	76	0	0,569	65	999	999	999	999	9	999	999	7	999	999
378	0	19	77	1	0,651	80	0,416	20	0,308	30	5	5	4	4	4	3
379	1	19	75	0	1	85	0,874	80	1	85	5	5	5	0	0	0
380	0	19	73	0	0,824	95	0,755	65	0,591	60	5	7	7	5	4	6
381	1	19	83	1	0,813	70	0,824	90	0,813	70	5	6	5	2	3	2
382	0	19	71	0	0,769	90	0,7	75	0,769	80	7	7	8	0	1	0
383	1	19	80	1	0,293	60	0,651	70	0,399	70	8	9	7	6	8	4
384	0	19	81	0	0,805	60	0,781	65	0,805	70	7	8	7	2	4	2
385	1	19	81	0	0,843	60	0,597	50	0,597	80	5	11	8	4	5	4
386	0	19	76	1	0,689	85	0,805	999	0,867	80	1	1	1	1	1	0
387	0	19	78	1	0,569	50	0,359	55	0,441	50	6	9	7	6	8	6
388	0	20	84	0	1	90	999	999	999	999	3	999	999	0	999	999
389	1	20	76	1	0,693	70	0,707	60	0,798	85	4	3	4	3	3	2
390	0	20	80	1	0,69	75	0,781	55	0,781	50	8	4	7	5	4	5
391	1	20	73	0	0,833	85	0,805	85	0,707	90	3	7	7	0	0	0
392	0	20	74	0	0,775	70	0,833	90	999	999	4	999	4	3	999	2
393	1	20	74	1	0,749	40	999	999	999	999	10	999	999	4	999	999
394	0	20	75	0	1	100	1	85	0,874	85	2	0	0	0	0	0
395	1	20	72	1	0,807	90	999	999	999	999	8	999	999	3	999	999
396	0	20	71	0	0,7	80	0,73	50	0,71	55	11	9	6	2	3	5
397	1	20	90	1	0,806	85	0,638	65	0,687	80	2	3	7	0	0	0
398	0	20	80	0	1	100	0,917	100	1	95	0	2	1	0	0	0
399	1	20	84	1	0,837	80	0,781	55	999	999	6	4	999	2	2	999
400	0	20	77	1	0,308	60	0,334	65	0,432	65	9	6	5	6	5	5
401	1	20	76	0	0,897	80	1	80	1	80	0	0	0	0	0	0
402	0	20	73	0	0,73	80	0,737	86	999	999	7	8	999	2	0	999
403	1	20	76	1	0,876	90	0,912	90	1	80	0	0	0	0	0	0
404	0	20	85	0	0,775	50	999	999	999	999	7	999	999	1	999	999
405	1	20	71	1	0,689	70	0,651	55	0,683	60	9	9	10	4	3	5
406	0	20	73	1	0,698	70	0,71	40	0,753	40	4	4	2	3	3	2
407	1	20	91	0	0,116	40	0,317	70	0,248	80	3	3	3	3	2	2
408	1	20	91	1	0,799	80	0,54	90	0,7	90	5	5	4	3	4	2
409	0	20	81	1	0,719	65	0,689	65	0,713	60	4	7	3	3	3	2
410	1	20	87	1	0,668	80	0,632	80	0,785	75	8	8	1	1	2	1
411	1	21	79	1	0,378	40	999	999	0,369	40	10	9	9	8	8	9
412	0	21	86	0	0,912	70	999	999	999	999	3	999	999	3	999	999
413	1	21	83	1	0,874	75	0,874	85	1	90	2	1	2	0	0	0
414	0	21	85	1	0,584	60	0,514	66	0,807	65	7	10	8	5	4	6
415	1	21	76	1	0,781	60	0,677	70	0,651	65	6	5	6	6	5	6
416	0	21	75	0	0,683	85	0,833	85	0,781	45	6	6	5	1	1	1
417	1	21	88	0	0,775	55	0,788	50	999	999	5	6	999	4	6	999
418	0	21	85	0	0,608	60	0,35	65	0,681	65	6	7	10	5	5	5
419	1	21	82	1	0,843	80	1	70	1	77	1	0	0	1	0	0
420	0	21	77	1	0,597	70	0,719	65	0,781	40	6	6	7	3	2	3
421	1	21	88	1	0,498	50	0,498	60	0,694	70	7	7	5	6	4	4
422	1	22	81	0	0,733	70	0,874	80	0,765	85	7	6	4	0	0	0
423	0	22	87	1	1	90	0,912	85	0,912	80	3	5	6	0	0	0
424	1	22	75	0	0,845	90	0,845	65	0,845	70	3	6	4	0	1	1
425	0	22	72	1	0,874	70	0,813	75	0,833	70	9	9	7	0	1	0
426	1	22	80	1	0,755	70	0,766	70	0,677	70	4	3	6	2	1	2
427	0	22	90	0	0,912	90	0,805	80	0,876	90	9	7	5	0	0	0
428	0	22	76	0	0,912	80	0,912	85	0,833	80	2	2	3	2	0	1
429	1	22	84	0	0,833	90	0,912	80	1	90	4	3	1	1	0	0
430	0	22	72	1	0,876	90	0,912	80	1	95	5	5	4	0	0	0
431	1	22	72	0	0,781	75	0,64	65	0,689	85	5	6	4	0	2	1
432	0	22	83	1	0,813	50	0,781	40	0,7	60	10	10	12	6	4	8
433	1	22	74	1	0,595	60	0,737	55	0,583	50	10	8	10	8	0	5
434	0	22	76	0	0,73	50	0,781	85	0,781	85	5	6	9	4	4	4
435	0	22	81	1	0,775	70	0,775	40	0,565	70	3	4	3	2	3	3
436	1	22	76	1	0,833	85	0,763	90	0,737	70	4	5	6	0	0	0
437	0	22	72	0	0,765	90	0,874	95	0,874	85	2	1	1	0	0	0
438	1	22	80	0	0,792	80	0,792	75	0,775	85	4	4	9	4	3	3
439	0	22	75	1	0,775	60	0,807	70	0,792	70	6	4	3	1	0	0
440	1	22	93	1	0,435	60	0,742	75	999	999	5	3	999	0	1	999
441	0	22	89	0	0,713	80	0,749	60	0,667	60	4	4	9	1	3	6
442	1	22	84	1	0,874	90	1	90	1	90	2	1	10	0	0	0
443	0	22	84	1	0,837	75	0,817	65	0,683	70	4	7	4	0	0	4
444	1	22	78	0	0,7	75	0,707	85	0,795	85	6	7	5	3	1	0
445	0	22	85	0	0,7	70	999	60	0,632	75	8	7	8	0	2	0
446	1	22	77	1	0,805	70	0,707	80	0,876	75	9	9	6	4	3	0
447	0	22	75	1	0,799	60	0,743	80	0,799	60	3	2	3	1	2	1
448	1	22	71	1	0,677	60	0,719	65	0,677	55	8	8	9	6	4	6
449	0	22	71	0	0,837	75	0,805	70	0,837	70	6	3	3	1	1	0

450	1	22	78	1	0,308	40	0,446	999	0,075	15	10	9	12	7	6	9
451	1	22	84	1	0,683	70	0,657	999	999	999	7	3	999	1	0	999
452	1	23	75	0	1	90	0,912	80	1	75	1	1	0	0	0	0
453	0	23	77	1	0,736	90	0,715	80	999	999	2	999	4	0	999	1
454	1	23	77	0	1	80	0,415	60	999	75	3	6	3	2	5	0
455	0	23	87	1	0,775	85	0,681	80	0,651	999	10	9	9	10	8	6
456	1	23	84	0	1	90	1	95	1	94	3	3	1	0	0	0
457	0	23	92	1	0,27	15	0,589	50	0,083	70	7	4	9	5	2	4
458	1	23	77	1	1	100	1	100	999	999	1	1	999	0	0	999
459	0	23	78	1	0,805	80	0,781	45	0,584	55	5	6	5	3	3	4
460	1	23	84	1	0,275	50	0,394	50	0,394	60	7	7	7	5	3	2
461	0	23	85	1	0,775	90	0,766	75	0,781	80	6	7	7	4	5	5
462	1	23	75	0	0,833	90	0,833	80	0,912	80	3	2	0	3	0	0
463	0	23	75	1	0,805	75	0,805	75	999	999	2	999	1	1	999	0
464	1	23	86	1	0,788	70	0,707	80	0,707	75	11	10	10	0	0	0
465	0	23	77	0	0,912	70	0,833	80	0,912	70	2	4	2	0	1	1
466	1	23	78	1	0,687	55	0,677	70	0,321	50	5	6	7	3	4	2
467	0	23	76	1	0,516	50	0,208	45	0,451	35	6	6	8	3	5	5
468	1	24	75	1	0,805	70	0,805	75	0,799	60	8	8	8	6	6	4
469	0	24	80	1	0,296	20	999	999	999	999	4	999	999	2	999	999
470	1	24	89	1	0,707	75	0,681	70	0,503	60	5	5	5	2	4	4
471	0	24	72	1	0,775	75	0,707	60	0,781	60	6	6	5	4	5	4
472	1	24	80	0	0,726	80	0,726	85	0,726	70	3	4	10	3	2	2
473	0	24	84	0	0,625	70	999	75	999	999	5	3	999	3	2	999
474	1	24	74	0	0,833	80	0,876	95	0,876	85	5	4	4	4	0	3
475	0	24	72	0	1	90	1	85	1	50	0	1	0	0	0	0
476	1	24	72	1	0,749	55	0,385	50	0,35	30	12	11	10	7	5	5
477	0	24	76	0	1	80	1	90	0,912	85	1	2	2	1	0	0
478	1	24	86	1	0,628	60	0,806	50	999	999	7	6	999	1	6	999
479	0	24	72	0	0,793	80	0,713	70	0,785	60	5	5	3	3	4	2
480	1	24	76	0	0,912	75	0,833	75	0,805	80	3	2	5	0	1	1
481	0	24	81	0	1	95	0,833	80	1	95	1	4	2	0	0	0
482	1	24	88	1	0,912	70	1	75	0,912	70	4	3	4	0	0	0
483	0	24	76	0	1	80	1	85	1	80	3	1	1	2	0	0
484	0	24	81	0	0,833	80	0,733	70	999	999	6	2	999	0	2	999
485	0	24	72	0	1	80	1	85	1	80	0	0	1	0	0	0
486	1	25	84	1	0,503	10	0,503	60	0,489	65	7	6	5	7	3	2
487	0	25	74	0	0,833	90	0,707	80	0,707	70	7	11	10	0	1	2
488	1	25	91	1	0,705	50	0,337	50	999	999	10	999	6	2	999	2
489	0	25	73	1	0,257	55	0,719	70	0,437	60	6	7	7	4	5	4
490	1	25	72	1	0,833	60	0,833	60	0,833	60	6	7	7	0	4	3
491	1	25	73	0	0,677	40	0,677	45	0,775	55	9	10	9	6	7	5
492	0	25	82	1	0,608	45	0,408	50	0,502	50	7	8	8	4	5	4
493	0	25	84	1	0,707	60	0,775	50	0,775	70	12	8	10	7	3	4
494	1	25	85	0	0,775	70	0,792	80	0,799	70	7	8	5	1	2	2
495	0	25	83	0	0,707	75	0,657	45	0,689	75	7	8	8	3	5	5
496	1	25	73	1	0,799	67	0,799	51	999	999	7	999	5	3	999	1
497	0	25	83	0	0,798	80	0,805	999	0,726	90	8	6	3	2	0	1
498	1	25	82	0	0,874	90	1	80	999	999	3	999	1	0	999	0
499	0	25	80	0	0,805	80	0,874	80	0,805	85	7	5	11	0	0	0
500	1	25	86	1	0,707	70	0,707	70	0,707	70	7	11	11	2	4	4
501	1	26	80	0	1	90	1	90	1	90	2	1	2	1	0	1
502	0	26	73	1	0,736	90	0,804	95	0,736	60	5	5	4	1	1	4
503	1	26	77	1	0,674	70	0,805	95	999	999	5	999	2	2	999	0
504	0	26	78	0	0,781	82	0,781	64	0,824	80	5	11	3	2	1	2
505	1	26	72	1	0,348	50	0,651	70	0,651	65	8	7	7	2	2	2
506	1	26	82	1	0,7	70	0,632	70	0,638	70	7	7	4	5	3	1
507	0	26	78	1	0,766	50	0,503	70	0,601	75	6	5	5	5	4	5
508	0	26	77	1	0,733	80	0,775	70	0,736	70	8	8	8	0	0	0
509	1	26	84	0	0,833	80	0,874	90	0,805	65	5	4	6	1	2	0
510	1	26	81	1	0,713	65	0,719	70	0,713	65	9	7	8	4	4	3
511	0	26	72	1	0,775	60	0,775	50	0,775	65	10	10	11	5	5	6
512	1	26	78	1	0,788	75	0,817	70	0,811	50	1	1	2	1	1	1
513	0	26	76	1	0,724	78	0,766	80	0,792	65	5	6	4	2	4	3
514	0	26	75	0	1	80	0,837	80	1	85	3	3	2	2	0	0
515	0	26	78	1	0,874	75	0,874	80	0,874	80	4	5	5	0	1	1
516	0	26	75	0	0,467	80	0,681	85	0,749	80	4	5	4	4	3	3
517	0	26	77	1	0,861	60	0,805	60	0,867	55	4	2	3	3	1	3
518	0	26	72	0	1	80	1	85	0,874	85	6	3	1	1	2	0
519	1	27	82	0	0,668	70	0,689	80	0,486	80	7	5	6	2	1	2
520	0	27	88	0	0,912	100	0,912	95	0,912	95	3	3	3	0	0	0
521	1	27	91	1	0,724	50	0,73	70	0,71	65	8	7	8	4	6	5
522	0	27	82	0	0,837	75	1	70	0,912	75	5	2	4	0	0	2
523	1	27	78	0	0,799	100	0,781	70	999	999	7	999	4	2	999	0
524	0	27	78	1	0,589	60	0,781	60	0,674	60	5	5	6	5	4	5

525	1	27	88	1	0,618	60	0,775	55	0,766	70	10	8	9	6	2	3
526	0	27	78	0	0,893	60	0,867	65	999	999	9	8	999	2	3	999
527	1	27	78	0	0,765	70	0,736	70	0,767	65	2	4	3	2	2	2
528	0	27	89	1	0,416	50	0,571	90	999	999	7	9	999	4	3	999
529	1	27	80	1	0,805	80	0,677	50	0,683	70	4	4	4	4	1	2
530	1	27	72	0	0,874	70	1	65	0,833	68	4	3	5	1	1	0
531	0	27	78	1	0,874	85	0,843	85	999	999	5	7	999	2	3	999
532	1	27	88	1	0,775	65	0,489	65	0,608	65	5	7	5	4	2	1
533	0	27	83	0	0,833	80	0,798	85	999	999	2	2	999	1	0	999
534	1	27	91	0	0,867	90	0,861	80	999	999	7	5	999	0	0	999
535	0	27	86	0	0,807	80	0,824	80	0,833	75	5	8	7	2	3	3
536	1	28	82	1	0,681	50	0,805	85	0,798	85	7	8	8	1	2	4
537	0	28	86	0	1	90	0,874	90	1	85	4	4	1	1	0	0
538	1	28	83	0	0,874	80	0,833	80	1	80	2	5	1	0	0	0
539	0	28	80	0	0,798	60	0,912	40	999	999	9	999	8	4	999	5
540	1	28	80	1	0,385	40	0,334	40	0,497	50	7	9	9	6	9	5
541	0	28	80	0	0,912	70	0,893	70	999	999	4	999	9	0	999	3
542	1	28	79	1	0,833	50	0,833	75	0,813	75	10	5	3	5	3	3
543	0	29	77	1	0,917	90	999	999	999	999	2	999	999	0	999	999
544	1	29	86	1	0,62	60	0,719	60	0,628	60	6	7	4	4	3	0
545	1	29	77	0	0,726	70	0,804	75	0,795	60	8	8	8	6	6	6
546	0	29	89	0	1	70	1	70	0,837	74	11	5	3	2	2	2
547	0	29	76	0	0,833	90	0,833	70	0,798	75	4	6	6	0	1	4
548	1	30	76	0	0,874	90	0,874	95	1	100	1	1	0	0	0	0
549	0	30	83	0	0,496	55	0,591	50	0,492	45	7	8	7	4	5	5
550	1	30	77	0	0,788	60	999	50	999	999	8	8	999	3	2	999
551	0	30	82	0	0,805	75	0,715	90	0,833	85	3	4	2	1	2	0
552	1	30	71	0	1	90	999	999	999	999	3	999	999	2	999	999
553	0	30	73	0	0,606	67	0,73	60	999	80	12	12	12	10	10	7
554	1	30	88	1	0,707	75	0,707	75	999	999	12	999	12	4	999	0
555	0	30	73	1	999	60	999	999	999	999	6	999	999	6	999	999
556	1	31	78	0	0,874	90	1	75	0,874	80	4	6	4	0	0	0
557	0	31	73	0	0,843	75	0,874	80	0,843	80	3	3	2	0	0	0
558	1	31	73	1	0,523	50	0,554	25	0,404	30	10	12	10	5	9	6
559	0	31	73	1	0,707	85	0,707	70	999	999	6	5	999	2	4	999
560	1	31	75	0	0,874	95	0,874	85	0,788	65	8	8	8	0	0	0
561	0	31	74	0	0,557	50	0,322	40	0,21	40	11	9	10	5	6	6
562	0	31	79	1	0,798	75	0,805	60	999	999	7	8	999	5	5	999
563	0	31	73	1	0,638	70	0,73	80	0,677	75	8	7	7	2	2	4
564	1	32	86	0	0,833	80	0,876	75	0,713	60	5	5	4	0	1	3
565	0	32	77	0	1	90	1	90	0,837	90	1	1	1	0	0	1
566	1	32	83	0	0,713	65	0,737	70	0,73	60	7	7	8	4	1	1
567	0	32	81	0	0,874	90	0,833	80	0,833	85	2	1	1	0	0	0
568	1	32	92	1	0,804	80	999	999	999	999	3	999	999	0	999	999
569	1	32	82	1	0,805	75	0,805	70	999	999	10	10	999	3	3	999
570	0	32	89	1	0,912	75	0,912	75	0,912	80	3	2	3	3	1	2
571	0	32	91	0	0,781	80	0,781	90	0,755	90	4	6	7	1	2	2
572	1	33	73	1	0,874	65	0,874	70	0,874	70	7	6	6	1	2	1
573	0	33	74	0	0,861	100	0,861	100	0,766	80	1	3	2	1	2	1
574	1	33	71	0	0,798	90	0,799	70	0,798	70	5	4	3	1	1	1
575	0	33	76	1	0,565	50	0,749	35	0,565	50	3	4	3	3	1	2
576	1	33	71	1	0,845	60	0,737	60	999	70	4	5	5	4	4	4
577	0	33	70	0	0,874	85	0,874	85	0,874	85	2	2	2	0	0	0
578	1	33	80	1	0,833	70	999	999	999	999	5	999	999	1	999	999
579	0	33	79	0	0,874	75	0,833	80	0,813	65	1	3	5	0	0	1
580	1	33	82	0	0,798	80	0,805	85	0,876	80	5	7	5	0	0	0
581	0	33	71	0	0,813	80	0,781	60	0,874	95	2	1	2	2	1	0
582	1	33	78	1	0,583	50	0,543	40	0,595	55	12	12	10	5	6	5
583	0	33	69	1	0,807	75	0,874	75	0,833	70	3	2	4	0	0	0
584	1	34	81	0	0,893	55	0,876	85	0,861	75	1	2	3	1	2	0
585	1	34	78	1	0,296	50	0,464	65	0,435	65	5	4	4	3	3	3
586	0	34	80	1	0,129	20	0,275	30	0,32	80	5	7	7	5	7	7
587	1	34	83	1	0,713	48	0,73	50	0,799	70	11	11	10	8	3	7
588	0	34	77	0	0,867	60	0,793	70	0,799	60	1	1	3	1	1	2
589	0	34	72	0	0,837	75	0,828	60	0,805	60	6	5	4	0	0	0
590	1	34	87	1	0,876	78	999	90	0,781	80	3	4	2	0	0	0
591	0	34	84	0	0,651	50	999	999	999	999	8	999	999	5	999	999
592	1	34	71	0	1	90	1	100	1	100	0	0	0	0	0	0
593	0	34	75	1	0,798	80	0,805	60	0,805	85	4	6	4	2	2	3
594	1	34	88	0	1	98	1	95	0,912	95	0	1	1	0	0	0
595	0	34	87	1	0,707	50	999	75	0,713	60	6	6	7	4	3	6
596	1	34	88	1	0,275	55	0,275	50	0,394	50	8	6	9	2	3	5
597	0	34	73	1	0,874	75	0,874	80	0,874	80	3	2	2	1	0	0
598	1	34	72	0	0,893	80	0,833	85	0,7	80	7	6	6	5	1	2
599	0	34	78	0	0,775	60	0,798	50	0,681	60	7	8	8	2	3	4

600	1	34	78	0	0,689	50	0,683	50	0,625	60	11	11	11	7	9	9
601	0	34	78	0	0,824	80	0,724	75	0,824	85	7	5	7	1	1	0
602	1	34	76	1	0,874	70	0,817	60	0,833	75	3	3	4	0	3	2
603	0	34	73	0	0,874	69	0,833	56	0,874	70	6	3	3	0	1	0
604	1	34	83	0	0,845	95	1	90	0,833	95	5	2	6	1	0	0
605	0	34	71	0	0,833	80	0,813	75	0,807	70	2	4	4	0	0	2
606	1	34	84	0	0,833	90	0,833	70	999	999	1	0	999	0	0	999
607	0	34	79	1	0,837	80	0,828	70	1	75	4	3	1	0	1	0
608	1	34	78	0	0,765	90	0,765	95	0,874	90	4	2	1	0	0	0
609	0	34	80	0	0,792	80	0,798	64	0,798	70	4	4	4	2	2	1
610	1	34	78	1	0,651	60	0,651	55	0,651	55	11	8	9	8	7	8
611	0	34	80	1	0,707	70	0,737	75	999	999	7	7	999	0	4	999
612	1	34	82	0	0,798	70	0,781	60	0,775	60	7	9	8	2	4	2
613	0	34	86	0	0,833	80	0,805	80	0,805	80	4	3	3	1	0	1
614	0	34	84	0	0,874	80	999	999	999	999	6	999	999	0	999	999
615	0	34	87	1	0,733	70	0,745	70	0,733	85	4	3	3	0	1	0
616	0	34	87	0	0,781	50	0,781	60	0,781	60	6	5	9	4	3	6
617	1	35	77	0	0,781	80	0,683	65	999	999	4	7	999	4	2	999
618	0	35	76	1	0,677	50	0,359	40	0,651	60	7	8	8	5	8	7
619	1	35	80	1	0,917	60	0,917	60	0,917	75	2	2	2	0	1	0
620	0	35	73	0	0,713	70	0,713	70	0,707	80	7	8	9	7	8	7
621	1	35	83	1	0,707	65	0,71	75	0,707	50	9	8	11	4	1	3
622	1	35	75	0	0,425	100	0,408	70	0,355	80	1	3	2	1	2	2
623	0	35	78	0	0,394	60	0,27	50	999	999	4	1	999	2	0	999
624	1	35	81	0	1	90	0,897	80	0,829	70	1	1	2	0	0	1
625	0	35	88	0	0,874	80	0,874	80	0,833	80	2	2	5	0	0	0
626	1	35	82	1	0,689	999	999	70	0,689	60	5	5	7	2	2	2
627	0	35	74	1	0,693	60	0,608	70	0,775	65	5	5	6	4	4	4
628	1	35	75	0	0,833	75	0,677	50	999	70	9	9	10	2	1	2
629	1	35	73	0	0,798	85	999	75	0,788	75	5	5	6	2	2	4
