# Supplementary material for: Effects of a clinical medication review focused on personal goals, quality of life, and health problems in older persons with polypharmacy: A randomised controlled trial (DREAMeR-study)
Source: PLoS Med. 2019 May 8;16(5):e1002798. doi: 10.1371/journal.pmed.1002798 (PMC6505828; doi:10.1371/journal.pmed.1002798)
Supplement: S1 Table — (DOCX) [file pmed.1002798.s005.docx]

**S1 Table: The 12 various type of health problems measured with the questionnaire**

| Health problems |
| --- |
| Pain |
| Itching |
| Dyspnoea |
| Problems with walking (mobility) |
| Dizziness |
| Sedation |
| Constipation/diarrhoea |
| Gastric problems (reflux or ulcer) |
| Cognition |
| Fatigue |
| Dry mouth |
| Incontinence |
