# Supplementary material for: Effects of a clinical medication review focused on personal goals, quality of life, and health problems in older persons with polypharmacy: A randomised controlled trial (DREAMeR-study)
Source: PLoS Med. 2019 May 8;16(5):e1002798. doi: 10.1371/journal.pmed.1002798 (PMC6505828; doi:10.1371/journal.pmed.1002798)
Supplement: S2 Table — (DOCX) [file pmed.1002798.s006.docx]

| **S2 Table: Main outcomes of the linear mixed model analysis for intervention group compared to control group for health-related quality of life and health problems (unadjusted regression scores)** | | | | | | |
| --- | --- | --- | --- | --- | --- | --- |
| **Outcome** | **Group** | | **Time** | | **Group * Time** | |
|  | β | 95% CI | β | 95% CI | β | 95% CI |
| **Health-related quality of life** | | | | | | |
| EQ-5D-5L, utility values | -4.7*10^-5^ | -0.033 to 0.033 | -0.0025 | -0.010 to 0.0053 | -0.0011 | -0.012 to 0.010 |
| EQ-VAS | -3.5* | -6.7 to -0.030 | -1.0* | -1.9 to -0.16 | +1.6** | 0.45 to 2.8 |
| **Health problems** | | | | | | |
| Total problems | +0.39 | -0.053 to 0.84 | -0.042 | -0.16 to 0.080 | -0.14 | -0.32 to 0.028 |
| Problems with impact | +0.18 | -0.19 to 0.0.54 | -0.013 | -0.12 to 0.090 | -0.17* | -0.31 to -0.021 |
| β coefficient and 95% CI for group (control vs. intervention group), time (per 3 months for HR-QoL and health problems), group by time interaction  *p<0.05, **p<0.01.  Abbreviations: CI = Confidence Interval; VAS = Visual Analogue Scale  Definition problem with impact = severity VAS-score ≥5 and influence on daily life: moderate, severe, extreme  NB. The estimators in the column: “group * time” show the main difference in effects between the intervention group vs. control group per three months for HR-QoL and health problems | | | | | | |
