# Supplementary material for: Effects of a clinical medication review focused on personal goals, quality of life, and health problems in older persons with polypharmacy: A randomised controlled trial (DREAMeR-study)
Source: PLoS Med. 2019 May 8;16(5):e1002798. doi: 10.1371/journal.pmed.1002798 (PMC6505828; doi:10.1371/journal.pmed.1002798)
Supplement: S3 Table — EQ, EuroQol. (DOCX) [file pmed.1002798.s007.docx]

**S3 Table: Number of participants with problems on the six domains of the EQ-6D at baseline and at six months: comparison between control and intervention groups**

| **Outcome** | **Control group** | | **Intervention group** | | |
| --- | --- | --- | --- | --- | --- |
| **EQ-6 Domains** | Baseline  (n=314) | At 6 months (n=261) | | Baseline  (n=315) | At 6 months (n=266) |
| Mobility | 76% | 79% | | 77% | 78% |
| Self-care | 25% | 27% | | 27% | 26% |
| Daily activities | 63% | 66% | | 64% | 69% |
| Pain/other complaints | 79% | 79% | | 77% | 76% |
| Mood/anxiety | 30% | 27% | | 31% | 32% |
| Cognition | 52% | 52% | | 59% | 58% |
| Percentage of patients with light to severe problems on each domain (score 2-5) compared to no problems (score 1) | | | | | |
