# Supplementary material for: Effects of a clinical medication review focused on personal goals, quality of life, and health problems in older persons with polypharmacy: A randomised controlled trial (DREAMeR-study)
Source: PLoS Med. 2019 May 8;16(5):e1002798. doi: 10.1371/journal.pmed.1002798 (PMC6505828; doi:10.1371/journal.pmed.1002798)
Supplement: S4 Table — (DOCX) [file pmed.1002798.s008.docx]

| **S4 Table: Main outcomes of the linear mixed model analysis for intervention group compared to control group for health-related quality of life and health problems according to the per protocol analysis (n= 622 patients)** | | | | | | |
| --- | --- | --- | --- | --- | --- | --- |
| **Outcome** | **Group** | | **Time** | | **Group * Time** | |
|  | β | 95% CI | β | 95% CI | β | 95% CI |
| **Health-related quality of life** | | | | | | |
| EQ-5D-5L, utility values | 0.0078 | -0.025 to 0.041 | -0.0024 | -0.010 to 0.0054 | -0.0011 | -0.012 to 0.010 |
| EQ-VAS | -3.0 | -6.2 to -0.13 | -1.0* | -1.9 to -0.18 | +1.7** | 0.49 to 2.9 |
| **Health problems** | | | | | | |
| Total problems | +0.43 | -0.094 to 0.96 | -0.044 | -0.17 to 0.080 | -0.14 | -0.32 to 0.030 |
| Problems with impact | +0.26 | -0.17 to 0.70 | -0.015 | -0.12 to 0.090 | -0.17* | -0.31 to -0.019 |
| β coefficient and 95% CI for group (control vs. intervention group), time (per 3 months for HR-QoL and health problems), group by time interaction (adjusted for age, sex, pharmacy)  *p<0.05, **p<0.01.  Abbreviations: CI = Confidence Interval; VAS = Visual Analogue Scale  Definition health problem with impact = severity VAS-score ≥5 and influence on daily life: moderate, severe, extreme  NB. The estimators in the column: “group * time” show the main difference in effects between the intervention group vs. control group per three months for HR-QoL and health problems | | | | | | |
