# Supplementary material for: Effects of a clinical medication review focused on personal goals, quality of life, and health problems in older persons with polypharmacy: A randomised controlled trial (DREAMeR-study)
Source: PLoS Med. 2019 May 8;16(5):e1002798. doi: 10.1371/journal.pmed.1002798 (PMC6505828; doi:10.1371/journal.pmed.1002798)
Supplement: S5 Table — (DOCX) [file pmed.1002798.s009.docx]

**S5 Table: Top 10 most frequently added and ceased drugs in both groups classified on ATC-5 level**

|  | **Added** | **CG** | **IG** |  | **Ceased** | **CG** | **IG** |
| --- | --- | --- | --- | --- | --- | --- | --- |
| **ATC-5** | **Drug class** | **n** | **n** | **ATC-5** | **Drug class** | **n** | **n** |
| A11CC | Vitamin D | 11 | 41 | A02BC | Protonpump inhibitors | 12 | 23 |
| N02BE | Anilides (paracetamol) | 10 | 23 | B01AC | Platelet aggregation inhibitors | 20 | 22 |
| A02BC | Protonpump inhibitors | 11 | 21 | S01XA | Other ophthalmologicals | 5 | 14 |
| S01XA | Other ophthalmologicals | 14 | 21 | C10AA | HMG CoA reductase inhibitors | 14 | 12 |
| A06AD | Osmotically acting laxatives | 21 | 20 | A06AD | Osmotically acting laxatives | 8 | 12 |
| R03AC | Selective beta-2-adrenoreceptor agonists | 6 | 14 | C08CA | Dihydropyridine derivatives | 11 | 7 |
| R01AD | Corticosteroids (nasal) | 14 | 8 | R03BB | Anticholinergics (inhalants) | 8 | 10 |
| C10AA | HMG CoA reductase inhibitors | 9 | 13 | C07AB | Beta blocking agents, selective | 10 | 5 |
| R03AK | Adrenergics and corticosteroids (inhalants) | 4 | 13 | N02BE | Anilides (paracetamol) | 10 | 8 |
| N02AA | Natural opium alkaloids | 13 | 11 | R03AC | Selective beta-2-adrenoreceptor agonists (inhalants) | 9 | 7 |
| Abbreviations: ATC = Anatomic Therapeutic Chemical classification; CG = control group; IG = intervention group; n = number of patient | | | | | | | |
