# Supplementary material for: Effects of a clinical medication review focused on personal goals, quality of life, and health problems in older persons with polypharmacy: A randomised controlled trial (DREAMeR-study)
Source: PLoS Med. 2019 May 8;16(5):e1002798. doi: 10.1371/journal.pmed.1002798 (PMC6505828; doi:10.1371/journal.pmed.1002798)
Supplement: S6 Table — (DOCX) [file pmed.1002798.s010.docx]

**S6 Table: Healthcare consumption over six months study period for the intervention group compared to control group**

| **Type of healthcare consumption** | **Control group (n=314)** | **Intervention group (n=315)** |
| --- | --- | --- |
| **Primary care** |  |  |
| Number of visits to GP | 821 | 904 |
| Number of visits to practice nurse | 326 | 324 |
| Number of visits to physiotherapist | 2225 | 1654 |
| Number of visits to psychologist, dietician or speech therapist | 186 | 156 |
| **Secondary care** |  |  |
| Number of visits to outpatient clinics | 739 | 620 |
| Number of visits to the emergency department | 44 | 46 |
| Number of visits to the hospital  (maximum of one day, mainly for investigation) | 110 | 217 |
| Hospital admissions, acute  (total number of days in hospital) | 202 | 166 |
| Hospital admissions, planned  (total number of days in hospital) | 43 | 55 |
| **Institutional care (rehabilitation, psychiatric or nursing home)** | | |
| Number of day visits | 119 | 20 |
| Total number of nights staying in the care home | 237 | 199 |
| **Homecare** |  |  |
| Average number of hours homecare per week  (including housekeeping and nursing) | 849 | 954 |
| **Informal care** |  |  |
| Total number of patients with an informal care giver | 107 | 103 |
