# Supplementary material for: Effects of a clinical medication review focused on personal goals, quality of life, and health problems in older persons with polypharmacy: A randomised controlled trial (DREAMeR-study)
Source: PLoS Med. 2019 May 8;16(5):e1002798. doi: 10.1371/journal.pmed.1002798 (PMC6505828; doi:10.1371/journal.pmed.1002798)
Supplement: S7 Table — DRP, drug-related problem. (DOCX) [file pmed.1002798.s011.docx]

**S7 Table: Classification and solving of drug-related problems in the intervention group (n = 315)**

| DRP type | Number of identified DRPs | Percentage of solved DRPs |
| --- | --- | --- |
| Suboptimal therapy | 542 | 57% |
| Overtreatment | 335 | 59% |
| (potential) adverse effect | 272 | 73% |
| Drug not effective | 167 | 76% |
| Drug interaction | 21 | 86% |
| Contra-indication | 32 | 59% |
| Dose too high | 49 | 74% |
| Dose too low | 64 | 81% |
| Non-compliance | 38 | 100% |
| Inconvenience of use | 99 | 81% |
| Wrong dosage form | 45 | 87% |
| Other | 87 | 74% |
| Total | **1751** | **67%** |
| Abbreviations: DRP = drug-related problem.  NB. Data on DRPs was only available for 300 of 315 patients in the intervention group. | | |
