# Supplementary material for: Effects of a clinical medication review focused on personal goals, quality of life, and health problems in older persons with polypharmacy: A randomised controlled trial (DREAMeR-study)
Source: PLoS Med. 2019 May 8;16(5):e1002798. doi: 10.1371/journal.pmed.1002798 (PMC6505828; doi:10.1371/journal.pmed.1002798)
Supplement: S1 Fig — (DOCX) [file pmed.1002798.s012.docx]

**Pain**

1. **Do you suffer from pain? O Yes O No**When yes, go to question 2 and 3
2. **What score would you give your pain on a scale from 0 – 10?**Cross the most appropriate number on the bar below


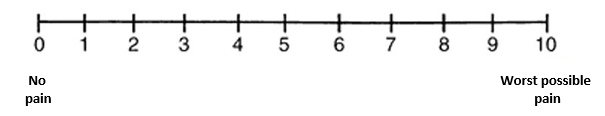


1. **To what extent does pain influence your daily life?**Cross the most appropriate answer below

**O Not at all O A little O Moderate O Severe O Extreme**

**S1 Fig: Example of the health problem type ‘pain’ as used in the questionnaire**
